# Phenotypic variation and genome-wide association studies of main culm panicle node number, maximum node production rate, and degree-days to heading in rice

**DOI:** 10.1186/s12864-022-08629-y

**Published:** 2022-05-23

**Authors:** Darlene L. Sanchez, Stanley Omar PB. Samonte, Jasper Benedict B. Alpuerto, Peyton A. Croaker, Karina Y. Morales, Yubin Yang, Lloyd T. Wilson, Rodante E. Tabien, Zongbu Yan, Michael J. Thomson, Endang M. Septiningsih

**Affiliations:** 1 Texas A&M AgriLife Research Center at Beaumont, Beaumont, Texas 77713 USA; 2 Bayer Research and Development Services (Bayer Crop Science), Chesterfield, Missouri 63017 USA; 3 Department of Soil and Crop Sciences, Texas A&M University, College Station, Texas 77843 USA

**Keywords:** Rice, Main culm panicle node number, Maximum node production rate, Degree days to heading, Genome-wide association study

## Abstract

**Background:**

Grain yield is a complex trait that results from interaction between underlying phenotypic traits and climatic, edaphic, and biotic variables. In rice, main culm panicle node number (MCPNN; the node number on which the panicle is borne) and maximum node production rate (MNPR; the number of leaves that emerge per degree-day > 10°C) are primary phenotypic plant traits that have significant positive direct effects on yield-related traits. Degree-days to heading (DDTH), which has a significant positive effect on grain yield, is influenced by the interaction between MCPNN and MNPR. The objective of this research is to assess the phenotypic variation of MCPNN, MNPR, and DDTH in a panel of diverse rice accessions, determine regions in the rice genome associated with these traits using genome-wide association studies (GWAS), and identify putative candidate genes that control these traits.

**Results:**

Considerable variation was observed for the three traits in a 220-genotype diverse rice population. MCPNN ranged from 8.1 to 20.9 nodes in 2018 and from 9.9 to 21.0 nodes in 2019. MNPR ranged from 0.0097 to 0.0214 nodes/degree day > 10°C in 2018 and from 0.0108 to 0.0193 nodes/degree-day > 10°C in 2019. DDTH ranged from 713 to 2,345 degree-days > 10°C in 2018 and from 778 to 2,404 degree-days > 10°C in 2019.

Thirteen significant (*P* < 2.91 x 10^-7^) trait-single nucleotide polymorphism (SNP) associations were identified using the multilocus mixed linear model for GWAS. Significant associations between MCPNN and three SNPs in chromosome 2 (*S02_12032235, S02_11971745*, and *S02_12030176*) were detected with both the 2018 and best linear unbiased prediction (BLUP) datasets. Nine SNPs in chromosome 6 (*S06_1970442, S06_2310856, S06_2550351, S06_1968653, S06_2296852, S06_1968680, S06_1968681, S06_1970597,* and *S06_1970602*) were significantly associated with MNPR in the 2019 dataset. One SNP in chromosome 11 (*S11_29358169*) was significantly associated with the DDTH in the BLUP dataset.

**Conclusions:**

This study identifies SNP markers that are putatively associated with MCPNN, MNPR, and DDTH. Some of these SNPs were located within or near gene models, which identify possible candidate genes involved in these traits. Validation of the putative candidate genes through expression and gene editing analyses are necessary to confirm their roles in regulating MCPNN, MNPR, and DDTH. Identifying the underlying genetic basis for primary phenotypic traits MCPNN and MNPR could lead to the development of fast and efficient approaches for their estimation, such as marker-assisted selection and gene editing, which is essential in increasing breeding efficiency and enhancing grain yield in rice. On the other hand, DDTH is a resultant variable that is highly affected by nitrogen and water management, plant density, and several other factors.

**Supplementary Information:**

The online version contains supplementary material available at 10.1186/s12864-022-08629-y.

## Background

Grain yield is greatly influenced by interactions between underlying phenotypic traits and environmental variables. As a result, yield cannot be directly selected with a high degree of certainty. Selection must instead focus on traits that impact yield performance. In rice, tiller density and panicle mass-related traits, such as spikelet density, grain size, and grain number have been linked to with grain yield [1]. Genes that are associated with these traits have been identified, such as *IPA1*, *MOC1*, and *FC1* for tiller number [2–4], *APO1*, *DEP1*, and *Gn1a* for grain number [5–7], *GS3* for grain size [8], and *WFP* for panicle branching [9]. DNA markers for some of these genes have also been developed for use in trait selection [10, 11]. Studies have also shown that genes controlling heading date may influence grain yield in rice. QTLs for days to heading such as *Ghd7*, *Ghd7.1*, *Ghd8*, *Hd1*, *qHd1*, and *RFT1* have pleiotropic effects on yield-related traits in rice [12–19]. Interactions between QTLs for heading date also affect rice yield-related traits [20, 21]. Unfortunately, most measured rice phenotypic traits, including those mentioned above, are resultant variables that can be greatly impacted by several climatic, edaphic, biotic, and management variables. The further a resultant trait is from determining yield, the greater its variability and less its predictive value. In contrast, the closer a trait is to a genotype’s underlying yield response, herein referred to as a primary phenotypic trait, the less it is impacted by other variables and the greater its potential use as a selection criterion.

Process-based simulation modeling were used by our team to identify four primary traits that when combined putatively produce an ultra-high-yielding rice ideotype for the Gulf Coast environment in Texas, United States; namely, increased node production rate, increased main culm panicle node, increased leaf mass, and increased spikelet density [22]. These findings were verified in a field experiment, where 10 out of 10 rice genotypes with the combination of all four traits yielded more than the check cultivar, while three out of four genotypes that did not have all these four traits yielded less [23]. Using correlation and path analyses from field experiments, Samonte et al. [24] determined that main culm panicle node number (MCPNN) and maximum node production rate (MNPR) have significant direct effects on degree-days to heading (DDTH), mass per panicle, panicle density, and stem mass at heading and harvest, which are traits that have direct effects on grain yield.

Main culm panicle node number has been identified as an important biomass component since each culm node produces a leaf [25]. The average number of leaves that appear on the main stem per unit of thermal time has been suggested as an important constituent trait that explains genotypic variation of early vigor in rice [26]. This statistic is the inverse of the maximum node production rate when calculation is restricted to early season node production data. Similarly, the phyllochron has been used as an index in rice root and shoot development studies [27] and is suggested as a significant factor in predicting heading in rice [28]. The maximum phyllochron value when calculated prior to when node production begins to decrease as plant biomass rapidly increases is equal to the maximum node production rate when expressed on a heat unit basis.

Genome-wide association studies (GWAS) usually analyze diverse populations or individuals from various geographical locations or origins, as these have the advantage of capturing historical recombination events that occur during development [29]. Rice is an ideal plant species for GWAS, as its self-pollination and its long history of artificial selection have allowed the fixing of favorable alleles with large effects, and the diverse environments where rice is planted has resulted in rice subpopulations with distinctive combinations of traits that are adapted to respective local environments [30].

To understand the genetic basis of primary phenotypic traits MCPNN and MNPR, and resultant variable DDTH, this study was conducted with the following objectives: 1). to assess the phenotypic diversity of these traits in a panel of diverse rice accessions; 2). to determine genomic regions with which these traits are associated; and 3). to identify candidate genes involved with these traits.

## Results

### Phenotypic Variation in MCPNN, MNPR, and DDTH

Wide variation was observed among the three traits (Table 1). MCPNN ranged from 8.1 to 20.9 nodes in 2018 and from 9.9 to 21.0 nodes in 2019. MNPR ranged from 0.0097 to 0.0214 nodes/degree-day > 10°C in 2018 and from 0.0108 to 0.0193 nodes/degree-day > 10°C in 2019. DDTH ranged from 713 to 2,345 degree-days > 10°C in 2018 and from 778 to 2,404 degree-days > 10°C in 2019. Presidio, one of the check cultivars, had an average of 14.1 nodes on the main culm, 0.0156 nodes-degree day^-1^, and heading at 1300 degree-days in 2018. In 2019, Presidio had an average of 14.6 nodes on the main culm, 0.0144 nodes-degree day^-1^, and heading at 1384 degree-days. Some of the rice accessions were consistently in the tails of the trait distributions. The accession C4-63 had the highest MCPNN in 2018 and 2019, while accessions Csornuj and Short Grain were the earliest and latest, respectively, in terms of DDTH in both years. Broad-sense heritability (H^2^) estimates were high for MCPNN (0.83 in 2018 and 0.90 in 2019) and DDTH (0.99 in 2018 and 0.98 in 2019), while MNPR had low to moderate H^2^ estimates (0.20 in 2018 and 0.60 in 2019).

**Table 1:** Summary statistics and broad sense heritability (H^2^) estimates for main culm panicle node number (MCPNN), maximum node production rate (MNPR), and degree days to heading (DDTH) in 220 rice accessions evaluated at Texas A&M AgriLife Research at Beaumont in 2018 and 2019.

**Trait**	**2018**						**2019**					
	**Mean**	**Standard Deviation**	**Min**	**Max**	**Presidio (Check)**	**H**^**2**^	**Mean**	**Standard Deviation**	**Min**	**Max**	**Presidio (Check)**	**H**^**2**^
**MCPNN (nodes)**	14.2	1.9	8.1	20.9	14.1	0.83	15.2	1.8	9.9	21.0	14.6	0.90
**MNPR**^**a**^** (nodes/degree-day>10°C)**	0.0164	0.0023	0.0097	0.0214	0.0156	0.20	0.0151	0.0015	0.0108	0.0193	0.0144	0.60
**DDTH (degree-day>10°C)**	1,399	273	713	2,345	1,300	0.99	1,453	259	778	2,404	1,384	0.98

^**a**^Four outliers were removed

**Fig. 1:**
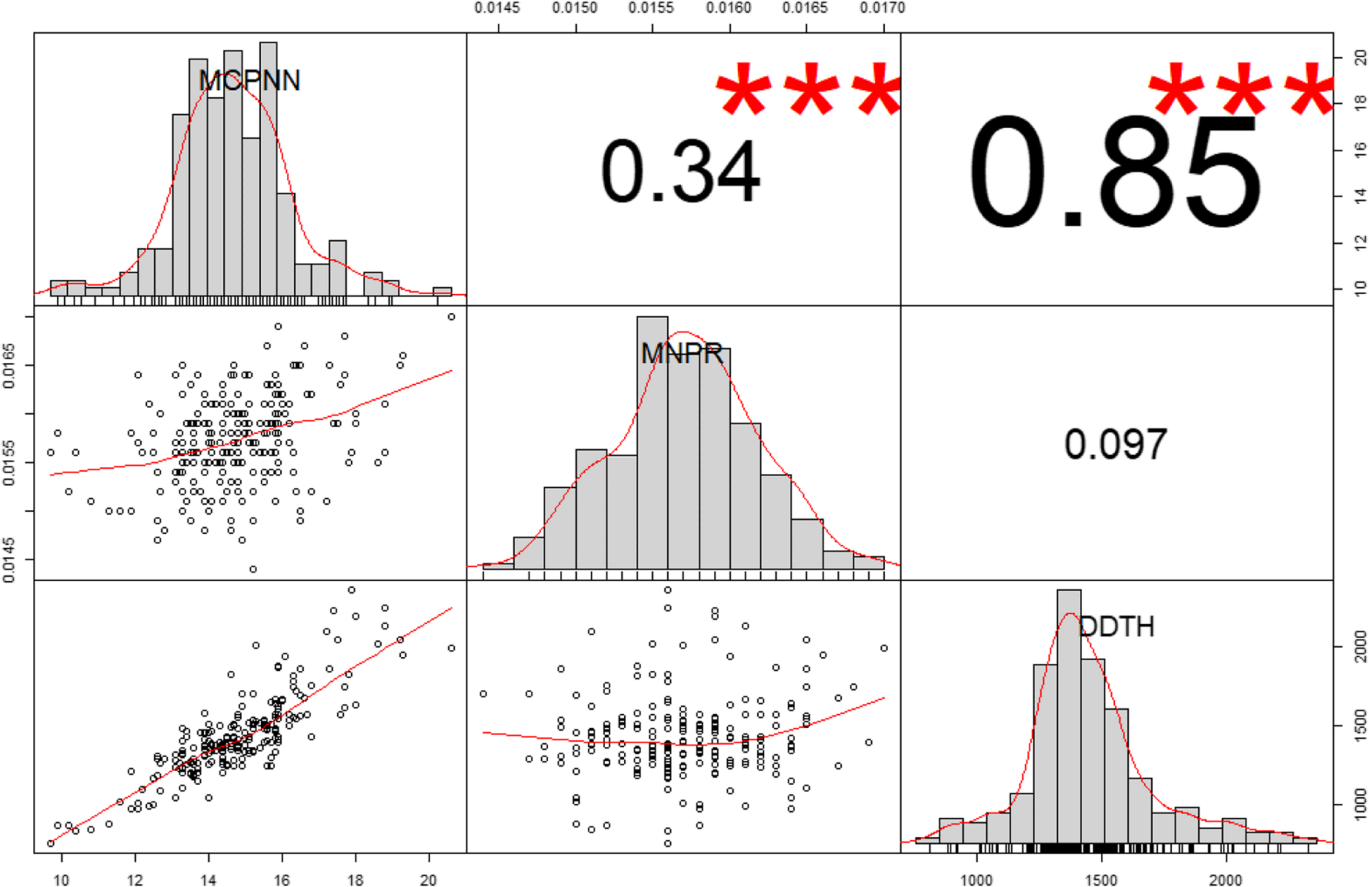
Frequency distribution and pairwise correlations of main culm panicle node number (MCPNN), maximum node production rate (MNPR), and degree days to heading (DDTH). The diagonal shows the histograms of the three traits. Shown below the diagonal are pairwise scatterplots. Shown above the diagonal are the pairwise Pearson correlation coefficients among MCPNN, MNPR, and DDTH. ***Significant at *p* = 0.001

**Fig. 2:**
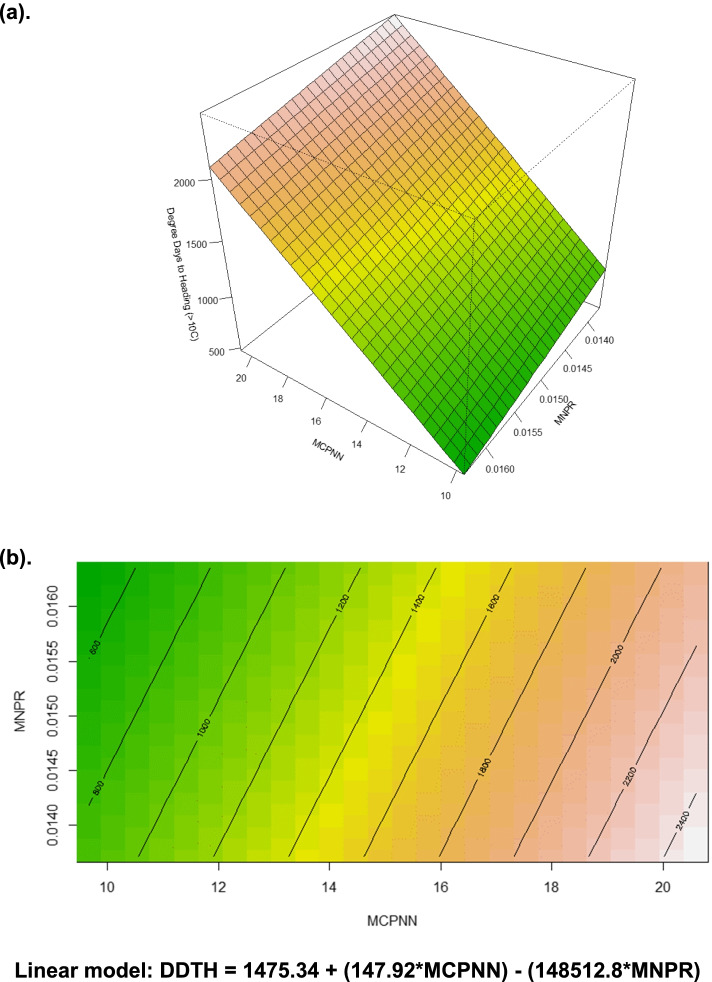
Perspective (**a**) and contour (**b**) plots showing the relationships of main culm panicle node number (MCPNN) and maximum node production rate (MNPR) on degree days to heading (DDTH) based on response surface methodology. The linear model is shown below the figure.

The three traits followed a normal or slightly positively skewed distribution (Fig. 1). Based on the BLUPs estimated from the two-years' data, MCPNN and DDTH had a very strong, positive correlation (Pearson correlation coefficient *(r*) = 0.85), MCPNN and MNPR had a moderately positive correlation (*r* = 0.34), and MNPR and DDTH had a weak positive correlation (*r* = 0.097) (Fig. 1). While frequency distribution trends for MCPNN and DDTH were similar in both years, MNPR had a slightly negatively-skewed distribution in 2018 and a normal distribution in 2019. Some variations in correlation coefficients of the three traits were observed in 2018 and 2019 (Supplementary Figure 1). MCPNN and MNPR had moderately positive correlations in 2018 (*r* = 0.29) and 2019 (*r* = 0.32), MCPNN and DDTH had a strong positive correlation in 2018 (*r* = 0.78) and a moderately positive correlation in 2019 (*r* = 0.34). MNPR and DDTH had a weak positive correlation in 2018 (*r* = 0.039) and a weak negative correlation in 2019 (*r* = -0.028). Analysis of variance (ANOVA) showed significant variation due to year and genotype effects for MCPNN and DDTH. For MNPR, significant variation due to year but no significant effect for genotype (*P* = 0.1509) was observed (Table 2). A response surface analysis shows both MCPNN and MNPR have highly significant effects (*P* < 0.0001) on DDTH [DDTH = 1475.34 + (147.92*MCPNN) - (148512.8*MNPR)], explaining 77.54% of the variation (Figure 2).

**Table 2:** Analyses of variance for main culm panicle node number, maximum node production rate, and degree-days to heading of rice accessions grown in Texas A&M AgriLife Research at Beaumont in 2018 and 2019.

**Trait: Main Culm Panicle Node Number**						
** Source**	**DF**	**Type III SS**	**Mean Square**	**F Value**	**Pr > F**	
** Year**	1	2.656059	2.656059	5.90	0.0218	*
** Block (Year)**	6	5.077442	0.846240	1.88	0.1198	
** Checks**	4	3.778924	0.944731	2.10	0.1077	
** Genotype**	214	1295.081128	6.051781	13.44	<.0001	***
** Year x Genotype**	207	173.741584	0.839331	1.86	0.0261	*
** Error**	28	12.608589	0.450307			
** Total**	461	1684.728905				
**Trait: Maximum Node Production Rate**						
** Source**	**DF**	**Type III SS**	**Mean Square**	**F Value**	**Pr > F**	
** Year**	1	0.00002941	0.00002941	10.84	0.0027	**
** Block (Year)**	6	0.00001973	0.00000329	1.21	0.3297	
** Checks**	4	0.00000872	0.00000218	0.80	0.5333	
** Genotype**	210	0.00079188	0.00000377	1.39	0.1509	
** Year x Genotype**	204	0.00052008	0.00000255	0.94	0.6149	
** Error**	28	0.00007598	0.00000271			
** Total**	454	0.00190784				
**Trait: Degree Days to Heading**						
** Source**	**DF**	**Type III SS**	**Mean Square**	**F Value**	**Pr > F**	
** Year**	1	437981.18	437981.18	17.32	0.0003	**
** Block (Year)**	6	90717.39	15119.56	0.60	0.7295	
** Checks**	4	132045.45	33011.36	1.31	0.2921	
** Genotype**	214	21341216.62	99725.31	3.94	<.0001	***
** Year x Genotype**	209	8100193.05	38756.90	1.53	0.0895	^.^
** Error**	28	708081.06	25288.61			
** Total**	463	30981213.13				

Levels of significance: *** *P* < 0.001, **P < 0.01, * *P* < 0.05, ^.^*P* < 0.1

### Genome-wide Association Studies and Identification of Candidate Genes

**Fig. 3:**
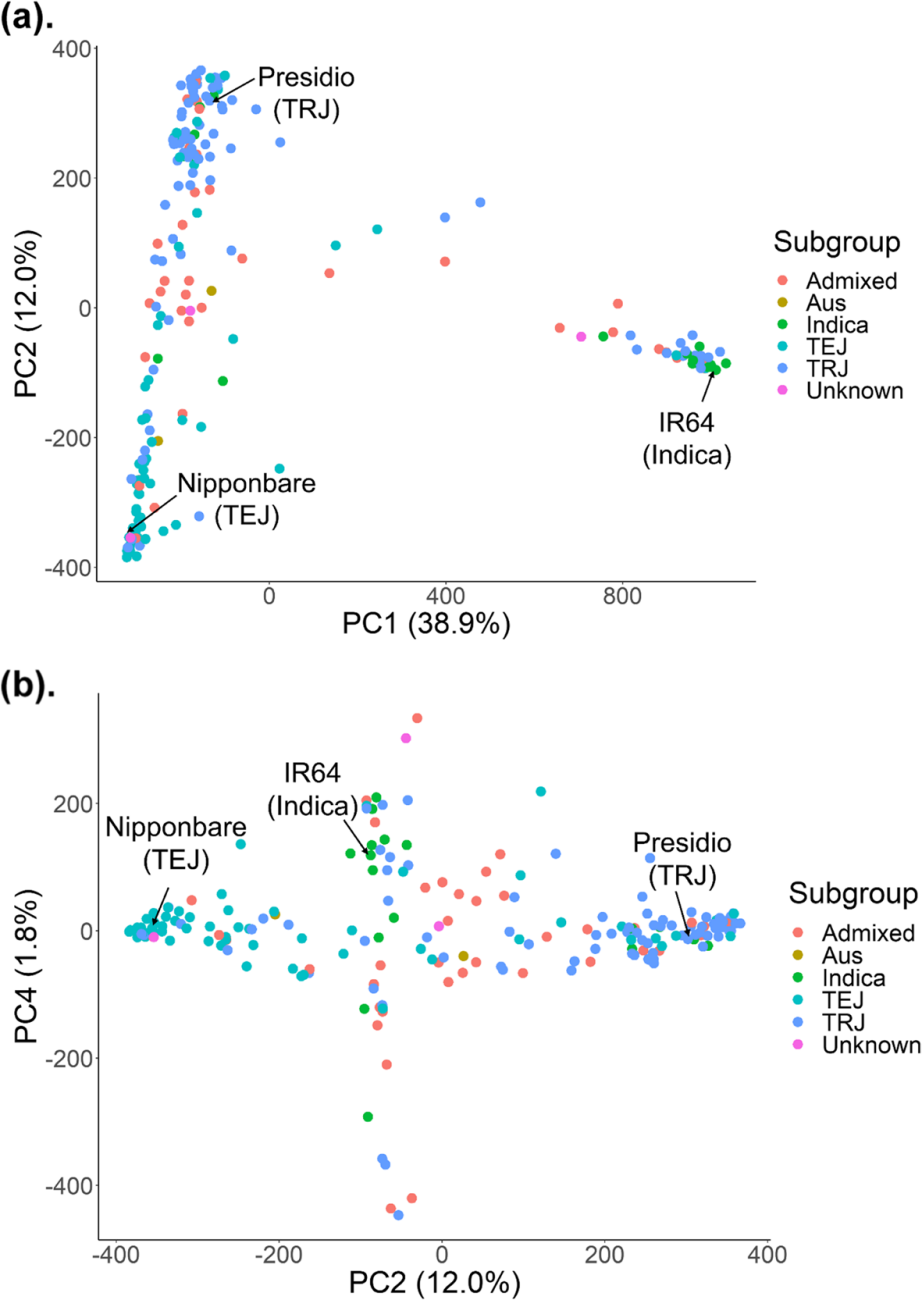
Principal component analysis of 220 diverse rice accessions used in genome-wide association studies (GWAS) for main culm panicle node number (MCPNN), maximum node production rate (MNPR), and degree days to heading (DDTH), showing (**a**) PC1 by PC2, and (**b**) PC2 by PC4. IR64, Nipponbare, and Presidio are labeled as representatives of the *indica*, *temperate japonica* (TEJ), and *tropical japonica* (TRJ) subgroups, respectively.

GWAS was conducted in the 220 accessions with both genotype and phenotype data. Because there was a significant effect of year on all three traits (Table 2), GWAS were conducted separately for the 2018 and 2019 data, as well as the BLUP estimates based on the data from both years. Principal component analysis (PCA) was used to determine population structure. The top four principal components (PCs) explained 56.7% of the genetic variation, wherein the first PC (38.9% of the variation) divided most of the *indicas* and *japonicas* into separate groups, while the second PC (12.0% of the variation) further separated the *japonica* group into *temperate japonicas* and *tropical japonicas*. The admixed accessions can be found in between these three major subgroups (Fig. 3).

Genome-wide linkage disequilibrium (LD) decay was estimated to be at ~150,000 bp. The results are within the range estimated in previous findings, ranged from close to 100,000 bp to over 200,000 bp [31–34].

**Fig. 4:**
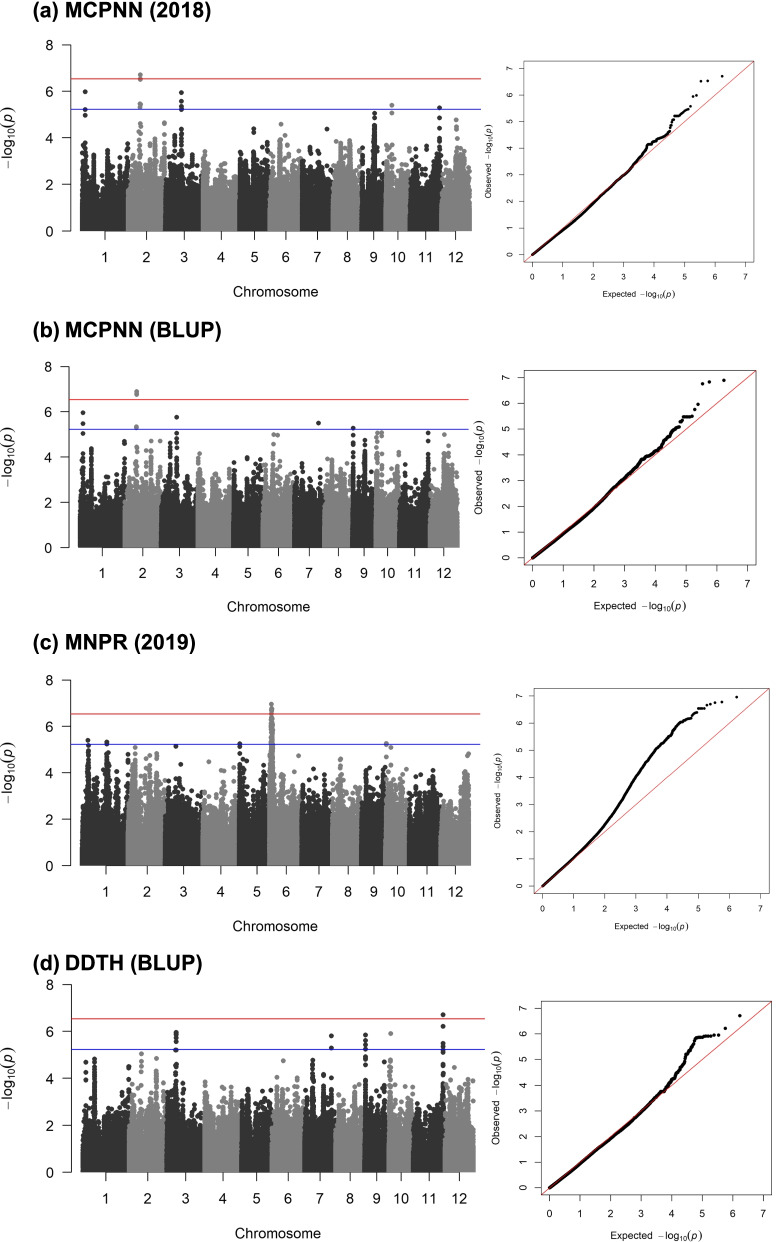
Manhattan and quantile-quantile (Q-Q) plots showing significant SNP-trait associations for (**a-b**) main culm panicle node number (MCPNN), (**c**) maximum node production rate (MNPR), and (**d**) degree days to heading (DDTH), detected using multilocus mixed linear model (MLMM). The red line denotes the genome-wide significance threshold (*P* = 2.91 x 10^-7^), and the blue line denotes the suggestive threshold (*P* = 5.83 x 10^-6^).

Thirteen significant trait-SNP associations were identified (Table 3 and Figure 4). Using the genome browser in RAP-DB (Nipponbare IRGSP Build 5), genes or gene models that include or are within 100 kilobase (kb) pairs to the SNPs declared to have significant associations with MCPNN, MNPR, and DDTH were identified (Supplementary Table 2). A total of 19 candidate genes were found to be in LD with the three SNPs significantly associated with MCPNN, 101 with the nine SNPs significantly associated with MNPR, and 21 with the SNP significantly-associated with DDTH.

**Table 3:** SNPs detected by GWAS using the multilocus mixed linear model (MLMM) as significantly associated with main culm panicle node number (MCPNN), maximum node production rate (MNPR), and degree days to heading (DDTH).

**Trait**	**SNP Marker**	**SNP Marker Location**		**Dataset**	**R**^**2**^	**P-value**	**Effect**
		**Chromosome**	**Position** **(base pairs)**				
**MCPNN (nodes) **	***S02_12032235***	2	12,032,235	2018	0.13	1.97 x 10^-7^	-1.3
	***S02_12032235***	2	12,032,235	BLUP	0.15	1.49 x 10^-7^	-1.1
	***S02_11971745***	2	11,971,745	BLUP	0.15	1.29 x 10^-7^	1.0
	***S02_12030176***	2	12,030,176	BLUP	0.15	1.74 x 10^-7^	-1.0
**MNPR (nodes/ degree day > 10°C)**	***S06_1970442***	6	1,970,442	2019	0.16	1.11 x 10^-7^	0.0013
	***S06_2310856***	6	2,310,856	2019	0.15	1.69 x 10^-7^	0.0012
	***S06_2550351***	6	2,550,351	2019	0.15	1.78 x 10^-7^	0.0012
	***S06_1968653***	6	1,968,653	2019	0.15	2.02 x 10^-7^	0.0012
	***S06_2296852***	6	2,296,852	2019	0.15	2.18 x 10^-7^	0.0013
	***S06_1968680***	6	1,968,680	2019	0.15	2.89 x 10^-7^	-0.0011
	***S06_1968681***	6	1,968,681	2019	0.15	2.89 x 10^-7^	-0.0011
	***S06_1970597***	6	1,970,597	2019	0.15	2.89 x 10^-7^	-0.0011
	***S06_1970602***	6	1,970,602	2019	0.15	2.89 x 10^-7^	0.0011
**DDTH (degree day > 10°C)**	***S11_29358169***	11	29,358,169	BLUP	0.14	1.94 x 10^-7^	-154

Significant association (*P* < 2.91 x 10^-7^, after multiple testing) between MCPNN and three SNPs in chromosome 2 (*S02_12032235, S02_11971745*, and *S02_12030176*) were detected in the 2018 and BLUP datasets. The SNP *S02_12030176* is within the *Oryza sativa nicotianamine aminotransferase 1 (OsNAAT1*) gene [35], in chromosome 2. The closest gene model to the SNP *S02_11971745* is Os02g0305600, described as a spectrin repeat-containing protein. Nine SNPs in chromosome 6 (*S06_1970442, S06_2310856, S06_2550351, S06_1968653, S06_2296852, S06_1968680, S06_1968681, S06_1970597,* and *S06_1970602*) were significantly associated with MNPR in the 2019 dataset. The SNP *S06_2296852* is within the *SULTR-like phosphorus distribution transporter* (*SPDT*) gene, also known as *Oryza sativa sulphate transporter 3;4* (*OsSULTR3;4*) [36, 37]. Three other SNPs associated with MNPR, *S06_1970442*, *S06_1970597*, and *S06_1970602,* are close to the candidate gene Os06g0137100 in the RAP-DP genome browser. The counterpart of Os06g0137100 in the MSU-RGAP genome browser [38], LOC_Os06g04560, is described as a kinesin motor domain-containing protein. In addition, candidate genes related to plant development, cell division and elongation, as well as protein and carbohydrate metabolism, were located within 100,000 bp of the identified SNPs for MCPNN and MNPR. One SNP in chromosome 11 (*S11_29358169*) was significantly associated with DDTH in the BLUP dataset. The closest candidate genes are Os11g0672300, which is described as being similar to a kinase domain-containing protein, and Os11g0672400, a calponin homology domain containing protein in chromosome 11.

## Discussion

### Phenotypic Analyses

Phenotyping MCPNN and MNPR is relatively time-consuming, compared to the typical measuring of resultant variables such as plant height, days to heading, days to peak flowering, and other secondary traits measured in most rice breeding programs. However, to state measuring a new trait is “time-consuming” is a value judgement based on a paradigm that assumes shallow phenotyping of large numbers of genotypes is the most efficient way to eliminate lines because they are suspected to result in poor yield performance. It might in fact be more cost-effective to spend more time measuring traits that have a greater influence on yield performance and possibly even evaluate fewer genotypes and thereby decrease the average amount of effort required to release a higher yielding genotype. A major goal of the current research is to identify relationships between the measured traits, and to develop markers to more efficiently select these traits.

There is wide variation in MCPNN, MNPR, and DDTH across the rice accessions. The standard deviations for MCPNN and MNPR are comparable to those of Samonte et al. [24], while that of DDTH was higher. The ranges for MNPR and the inverse of development rate observed by Rebolledo et al. [39], were similar to the estimates we obtained.

Broad-sense heritability (H^2^) estimates for the three traits ranged from 0.20 for MNPR (2018) to 0.99 for DDTH (2019). Modifying the planting method in 2019 (i.e., using jiffy pots and not thinning the plants before observation as was conducted in 2018) may have reduced plant stress, which may have improved estimation of the genotype effects, and thereby improved the H^2^ estimates for MNPR in 2019. However, because plant density impacts plant to plant competition, not thinning the plants could have also contributed to the observed differences comparing the years. The timing of N fertilization may have also affected the heritability estimated for MNPR, where two splits were done in 2018 (at planting and six weeks after planting), and three splits were done in 2019 (at planting, two weeks after planting, and 11 weeks after planting). Khing et al. [40] observed that phyllochrons are shortened (i.e., faster node production rate) with higher N rates or wider spacing. Martinez-Eixarch et al. [41] also reported that plant density and the timing of N fertilization, in combination with water management, affect the rate of leaf emergence. The total N fertilizer applied in both years were similar, hence, MCPNN and DDTH, which are considered as end-point traits, had consistently high heritability estimates.

The correlations between MCPNN, MNPR, and DDTH in the BLUP estimates from this study confirm the findings of Samonte et al. [24]. The pairwise correlation coefficients among MCPNN and MNPR (*r* = 0.34 in this study, and *r* = 0.35 in [24]) as well as MCPNN and DDTH (*r* = 0.85 in this study, and *r* = 0.86 [24]) were very close. The correlation between MNPR and DDTH was positive but low in both studies.

Samonte et al.[24] reported MCPNN and MNPR have significant positive and negative direct effects, respectively, on DDTH based on path analysis. In this study, response surface regression shows both MCPNN and MNPR have highly significant effects on DDTH (R^2^ = 0.78), and follow the same trend as reported by Samonte et al. [24]. The high degree of explained variability suggests the model can accurately predict DDTH relatively early in a genotype’s development.

### Genome-Wide Association Studies and Identification of Candidate Genes Related to MCPNN and MNPR

SNP-trait associations for MCPNN, MNPR, and DDTH were determined using the multi-locus mixed model approach, which included PCA for population structure and kinship matrix to minimize false associations and stepwise linear mixed-model regression with significantly-associated markers as cofactors. MCPNN was significantly associated with three SNPs in chromosome 2, which were detected in the BLUP dataset, which uses the data from both 2018 and 2019. In the individual experiments, only *S02_12032235* is significantly associated with MCPNN. In the 2019 dataset, while no significantly associated SNPs were detected, these three SNPs were in the top 20, with the peak at *S02_12032235*. The *OsNAAT1* gene harbors the SNP *S02_12030176* and is in high linkage disequilibrium (*r*^2^ = 0.91) with *S02_12032235*. *OsNAAT1* plays a role in iron (Fe) homeostasis in rice, where it is involved in Fe acquisition from the soil and transport within the plant [35, 42]. *OsNAAT1* also flanks a QTL for grain yield in chromosome 2 in rice [43]. The gene model Os02g0305600 is described as a spectrin repeat-containing protein and is 983 kb from *S02_11971745*. Spectrin-like proteins have been detected in the plant nuclei and plasma membranes and are suggested to perform multiple roles, such as stability and ion transport [44–46].

MNPR is significantly associated with *S06_2296852,* which lies within the *SPDT* (*OsSULTR3;4*) gene. *SPDT* is found to be highly expressed in node 1, which connects the panicle to the flag leaf, and serves as a switch to distribute phosphorus to the grains [37]. Three other SNPs significantly associated with MNPR, *S06_1970442*, *S06_1970597*, and *S06_1970602* are in high linkage disequilibrium (*r*^*2*^ = 0.73) with the gene model Os06g0137100. This gene model is similar to a predicted kinesin-like protein. Kinesins are microtubule motor proteins involved in cell division and growth. In rice, genes encoding kinesins confer traits related to plant height, grain length, and shape, and pollen partial sterility [47–53]. The gene model Os06g0137100 is also associated with grain number based on GWAS conducted by Huang et al. [33].

Some of the candidate genes within 100 kb of SNPs (Supplementary Table 2) are significantly associated with MCPNN and MNPR and are of interest as these have roles in plant development, cell division and elongation, as well as protein and carbohydrate metabolism. For instance, the gene models Os02g0305950 and Os06g0137400, which are close to the significantly-associated SNPs for MCPNN and MNPR, respectively, are putative small auxin-up RNA (SAUR) genes. SAUR genes encode auxin-responsive proteins that are primarily expressed in elongating tissues, and may play a role in regulating cell elongation [54].

*S11_29358169*, a SNP significantly associated with DDTH in the BLUP dataset, is in linkage disequilibrium (*r*^*2*^ = 0.96) with the gene model Os11g0672300, which is similar to a protein kinase domain-containing protein but needs to be characterized further to determine its specific function. In plants, the protein kinase superfamily is very broad and diverse, with roles in metabolic signaling, stress response, cell division regulation, and plant-specific functions, such as flowering [55].

Genes, QTLs, and chromosomal regions identified to be associated with MCPNN, MNPR, and DDTH in the literature were checked to determine if they overlap with the SNPs identified in this study. While none of the SNPs are co-located with reported or identified genes, some are close to previously reported QTL associated with heading date, such as *Hd17*, which is located at a 2.2 Mb region in chromosome 6 [56] and *qHD11.3*, located in a 28.7 Mb region in chromosome 11 [57]. Heuer et al. [58] reported that *ZmMADS3* in maize is involved in node number. In this study, there is no significant SNP-trait association in the region where the closest homolog of *ZmMADS3* in rice, *OsMADS15* in chromosome 7, is located. For MNPR, two regions in Chromosome 7 are significantly associated with node development rate [39], while QTLs for phyllochron have been mapped in chromosomes 1, 2, 4, 9, 10, and 11 [59, 60]. However, these regions do not contain any SNPs that are significantly associated with MNPR in this study.

## Conclusions

SNP markers significantly associated with MCPNN, MNPR, and DDTH were detected by association analyses using the multilocus linear mixed model. The identified SNPs are located within or in LD with gene models, which could be potential candidate genes for these traits. Further validation of the candidate genes through expression and gene editing analyses are needed to confirm if they are the causal agents for these traits. Identifying the genes regulating MCPNN and MNPR could lead to the development of fast and efficient approaches for their estimation, such as marker-assisted breeding or gene editing. Functional markers can be designed and could be used to select for early-generation breeding lines possessing higher MCPNN and faster MNPR. Gene editing can also be applied to alter the MCPNN and MNPR of a rice variety in order to achieve optimum yields. Understanding the underlying genetic basis for MCPNN, MNPR is an essential step in increasing breeding efficiency for enhancing grain yield in rice.

## Methods

### Plant Materials

A total of 220 rice accessions consisting of a diverse set of *indica* and *japonica* cultivars, landraces, inbred lines, and hybrids (Supplementary Table 1) were selected for their variation in degree-days to heading. These were planted at the Texas A&M AgriLife Research Center (Beaumont) in 2018 and 2019.

### Field Experiment Set-up

In 2018, the entries were drill-seeded on April 19 in three-row plots that were 2.44 m long, with rows spaced 0.25 m apart, using a randomized complete block design with 4 replications. A one-meter segment in the middle row of each plot was marked with flags and thinned to 15 plants, for data collection. Urea was applied in two splits: 108.4 kg ha^-1^ N at planting and 128.9 kg ha^-1^ N at 6 weeks after planting.

In 2019, the entries were sown on April 16-17 in three-row plots that were 2.44 m long, with rows spaced 0.28 m apart. The slight change in row spacing for the two years is due to the different planters used in each year. The side rows of each plot were mechanically drill-seeded, and the middle rows were manually planted at the same time as the drill-seeding. The middle row of each plot was created by digging a 2.44-m long furrow (approximately 6 cm deep), and a strip of eighteen 5.7 cm square x 5.7 cm deep biodegradable Jiffy pots (Jiffy International; www.jiffygroup.com) each with one seed placed in the center of each row. The Jiffy pots were marked with flags, while the ends of the middle rows were seeded with the designated entry using a manual seeder. This was done to eliminate thinning the plants before data collection. The field was flash-flooded on April 17, after the drill-seeding and manual planting were completed for all accessions. Fifteen of the 18 plants were used for data collection, with the remaining three plants as backup. Urea was applied in 3 splits for a more efficient distribution of N: 59.4 kg N ha^-1^ at planting (17 April 2019), 128.9 kg N ha^-1^ at two weeks after planting (23 May 2019), and 47.1 kg N ha^-1^ at 11 weeks after planting (3 July 2019).

### Phenotyping for MCPNN, MNPR, and DDTH

Data collection for MNPR and MCPNN are described in Appendix 1. The emergence of leaves on the main culms of 15 plants within a designated one-meter length was recorded every week, starting at the third leaf stage. MNPR was estimated through regression of the average leaf emergence data during the 3^rd^ to 7^th^ leaf stages with cumulative degree-days > 10℃ from planting, with degree-day accumulation estimated from ambient temperature recorded within 0.4 km of the research site. In 2018, MNPR was estimated using leaf count data starting at two weeks after thinning to minimize the effects of stress caused by thinning, while in 2019, thinning was not necessary because the plants were already spaced with the use of the Jiffy pots. Leaf counting was continued until heading, where MCPNN was estimated as the number of leaves on the main culm plus one for its panicle [24]. Days to 50% heading for each entry was estimated to occur when 50% of the panicles have exerted from tillers and used to estimate degree-days from sowing to 50% heading.

### Phenotypic Data Analyses

The combined analysis of both years of data was conducted using SAS Version 9.4 (SAS 2016) for Windows. Fixed and random effects were estimated using PROC MIXED in SAS software, and these were used to estimate best linear unbiased predictions (BLUPs) in R version 3.6.1 [61]. Analyses of variance and broad-sense heritability for the measured variables for each year were estimated using the 'augmentedRCBD' package [62] in R, with Pearson's pairwise correlation coefficients also calculated using R. The relationships between MCPNN and MNPR and the response variable DDTH was estimated using the 'rsm' package [63] in R.

### Marker Data

DNA was extracted from leaf tissues of 220 rice accessions (Supplementary Table 1), collected from the field at the Texas A&M AgriLife Research Center (Beaumont) in July 2018. Extraction was performed using standard protocol for leaf tissue with the Thermo Fisher Scientific KingFisher Flex (Thermo Fisher Scientific, Waltham, MA, USA). The DNA samples were sent to the Texas A&M AgriLife Genomics and Bioinformatics Service (TxGen) at College Station for genotyping-by-sequencing (GBS), with 1X coverage. The reference genome used was *Oryza sativa* ssp. *japonica* cultivar Nipponbare, International Rice Genome Sequencing Project (IRGSP) Build 5 [38]. The raw genotype data was filtered, selecting single nucleotide polymorphisms (SNPs) having less than 50% missing data and minimum allele frequency (MAF) >5%. After initial filtering, imputation was conducted using BEAGLE V4.0 [64] in 1,075,302 SNP markers. The genotype data was filtered a second time after imputation using TASSEL 5.2.61 [65], and 854,832 SNPs were used in the analyses after removing SNPs with less than 5% MAF and more than 5% missing data. Four genotypes were eliminated from the analysis as outliers due to the ratio of 2018 to 2019 MNPR rates being greater than the 75^th^ quartile or less than the 25^th^ quartile by more than 1.5 x (75^th^ quartile - 25^th^ quartile) [66].

### Genome-wide Association Studies

GWAS for MCPNN, MNPR, and DDTH were conducted using the phenotype data for 2018 and 2019 separately, as well as the BLUPs estimated from data collected in both years. Factors that may cause false trait-SNP associations (i.e., population structure (Q) and genetic relatedness (K)) were controlled using principal component analysis (PCA) and kinship matrix, respectively. The most probable number of subpopulations was determined by plotting the number of principal components (PC) against the variance explained by the PCs, and the optimum number of PCs was selected when the decrease in variance has reached a plateau. In this study, the total number of PCs used to account for population structure was four. The VanRaden kinship algorithm [67] was used to construct a kinship matrix. Both PCA and kinship matrices were generated using the R package Genomic Association and Prediction Integrated Tool (GAPIT), Version 3 [68, 69]. Linkage disequilibrium decay was used to estimate the appropriate resolution for association mapping, with a sliding window of 50 markers calculated using TASSEL 5.2.61. Mean r^2^ was then computed every 10,000 base pairs (bp), with linkage disequilibrium decay determined as the distance in bp wherein the average r^2^ decreased to half its maximum value.

Association analyses were conducted using the multilocus mixed model (MLMM) [70] implemented in GAPIT Version 3. MLMM uses stepwise linear mixed-model regression that includes significantly associated markers as cofactors, with Q and K to account for false positives. Multiple testing was accounted for using the statistical program 'simpleM' [71, 72] implemented in R, which calculates the number of independent tests (M_*eff_G*_). The M_*eff_G*_ was used to compute for the multiple testing threshold in a similar way as the Bonferroni correction method in which the significant threshold (α= 0.05) was divided by the M_*eff_G*_ or P = α/ M_*eff_G*_. For this study, the multiple testing threshold to declare significant association was set to P = 2.91 x 10^-7^. Manhattan and quantile-quantile (Q-Q) plots were constructed using the R package 'qqman' [73]. Identification of genes that contain the significant SNPs was achieved using the Nipponbare IRGSP Build 5 genome browser (https://rapdb.dna.affrc.go.jp/viewer/gbrowse/build5/) in the Rice Annotation Project Database (RAP-DB) [74] and Michigan State University (MSU) Rice Genome Annotation Project (RGAP) genome browser (http://rice.plantbiology.msu.edu/cgi-bin/gbrowse/rice/) [38].

## Supplementary Information

**Additional File 1.** Supplementary Figures

**Additional File 2.** Supplementary Tables

**Additional File 3.** Appendix 1. Phenotyping for Maximum Node Production Rate (MNPR) and Main Culm Panicle Node Number (MCPNN)

## Data Availability

All the data supporting the results of this article are provided within the article or in the additional files. The genotyping data has been deposited at Dryad (https://doi.org/10.5061/dryad.4qrfj6qbs).
