# Supplementary material for: Phenotypic variation and genome-wide association studies of main culm panicle node number, maximum node production rate, and degree-days to heading in rice
Source: BMC Genomics. 2022 May 23;23:390. doi: 10.1186/s12864-022-08629-y (PMC9125873; doi:10.1186/s12864-022-08629-y)
Supplement: Supplementary file 1 — Additional File 1. Supplementary Figures [file 12864_2022_8629_MOESM1_ESM.docx]

1. **2018**


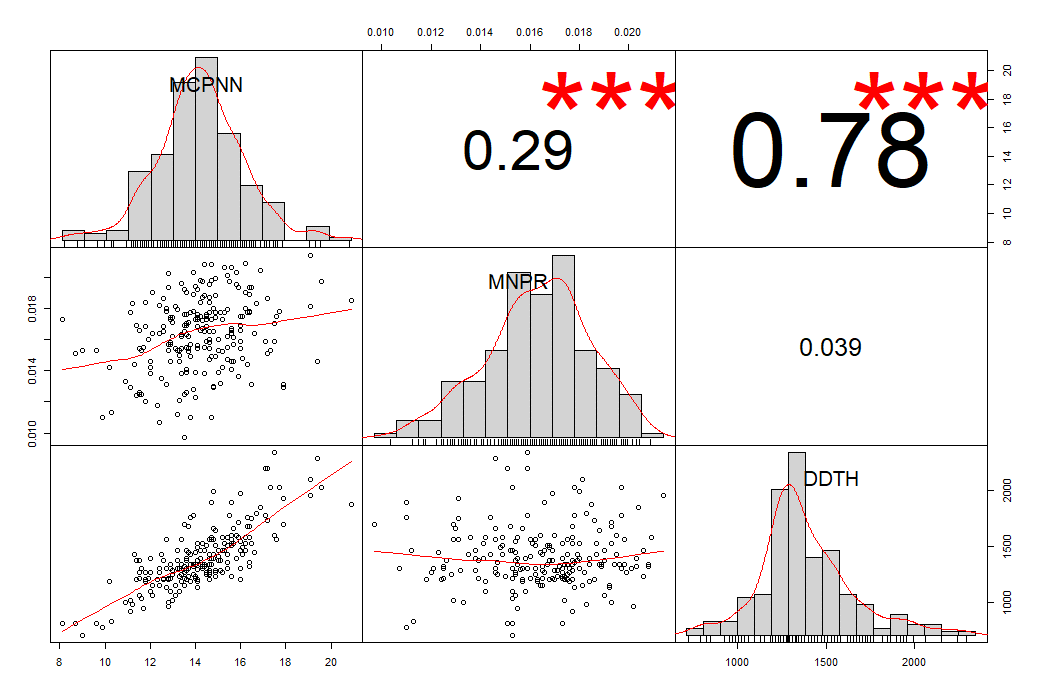


1. **2019**


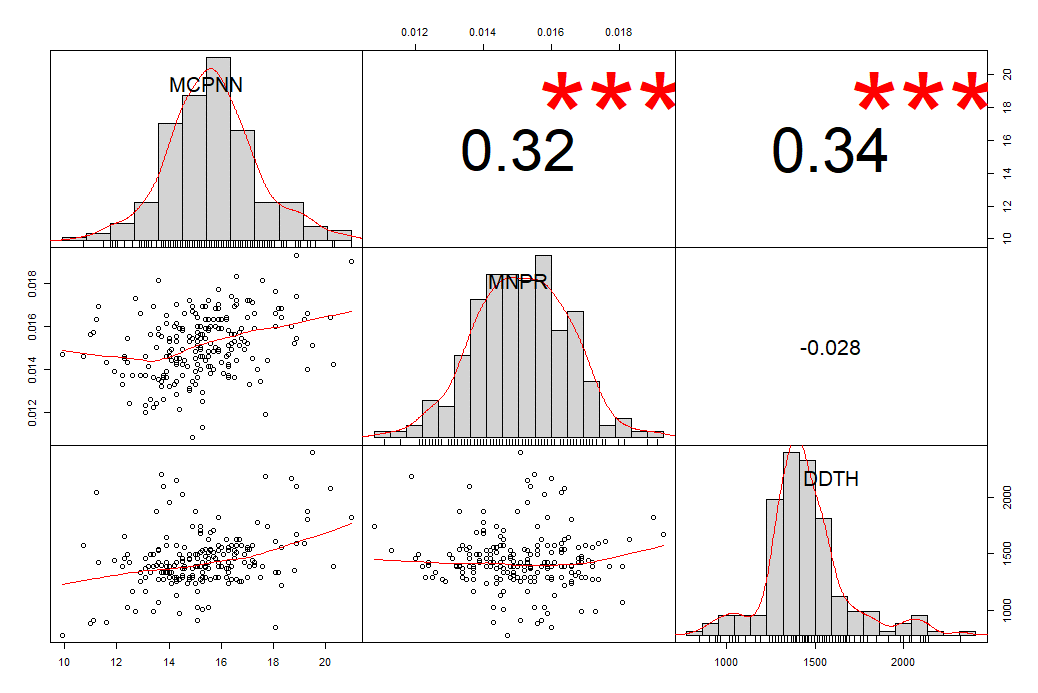


**Supplementary Figure 1**. Frequency distribution and pairwise correlations of main culm panicle node number (MCPNN), maximum node production rate (MNPR), and degree-days to heading (DDTH) of the rice population grown in Texas A&M AgriLife Research at Beaumont in (a) 2018 and (b) 2019. The diagonal shows the histograms. Shown below the diagonal are pairwise scatterplots. Shown above the diagonal are the pairwise Pearson correlation coefficients among MCPNN, MNPR, and DDTH. ***Significant at p = 0.001.

1. **MCPNN (2019)**


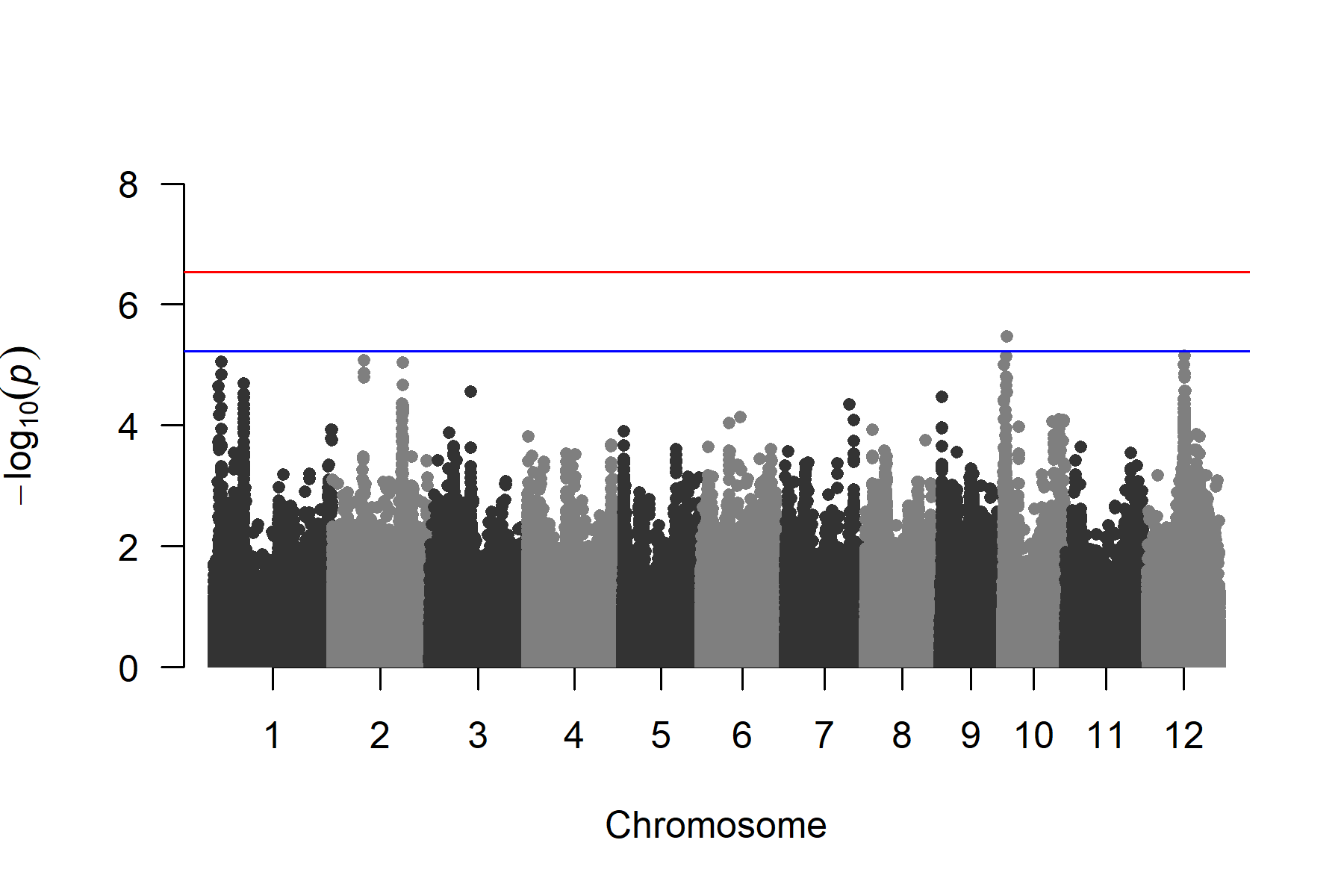

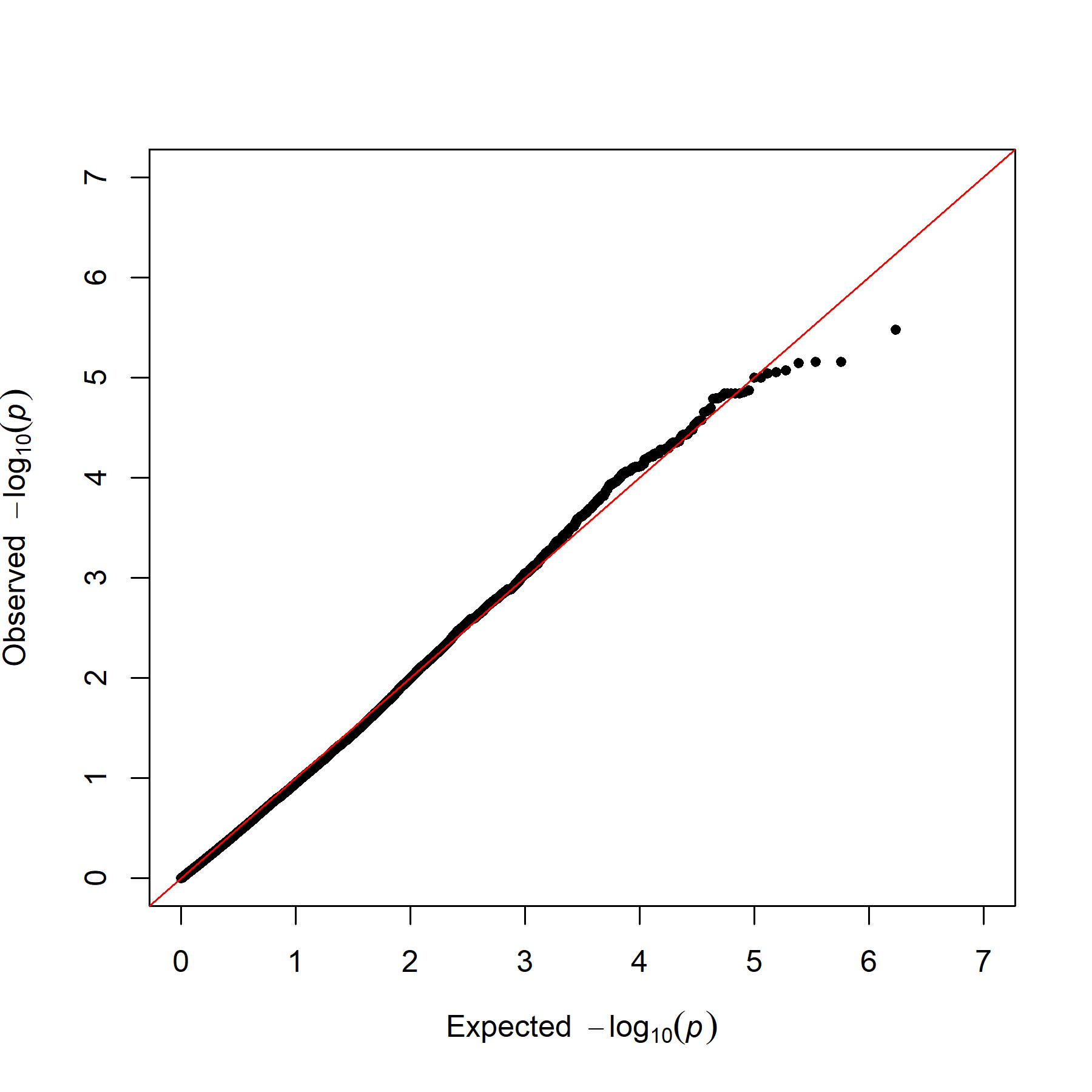


1. **MNPR (2018)**


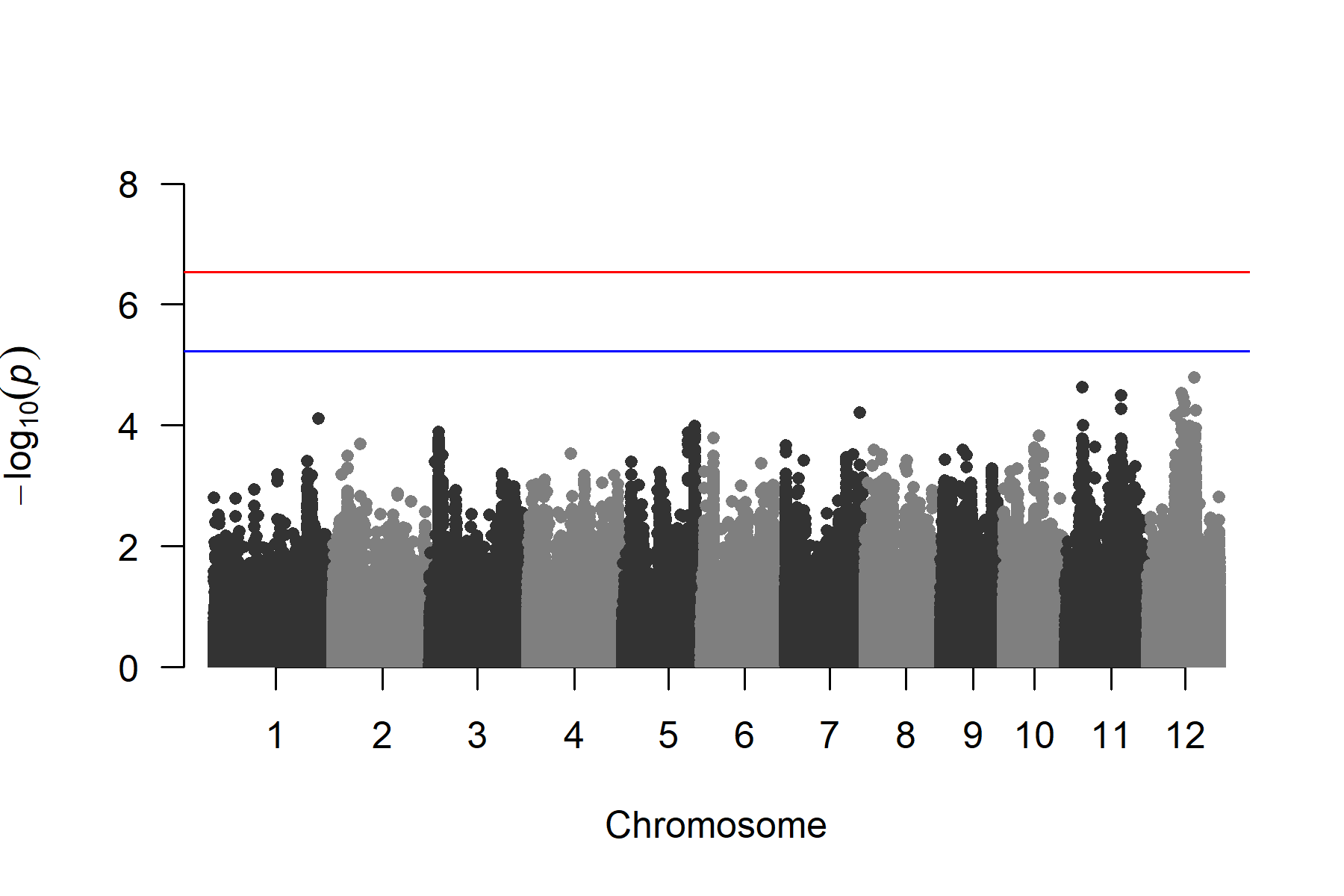

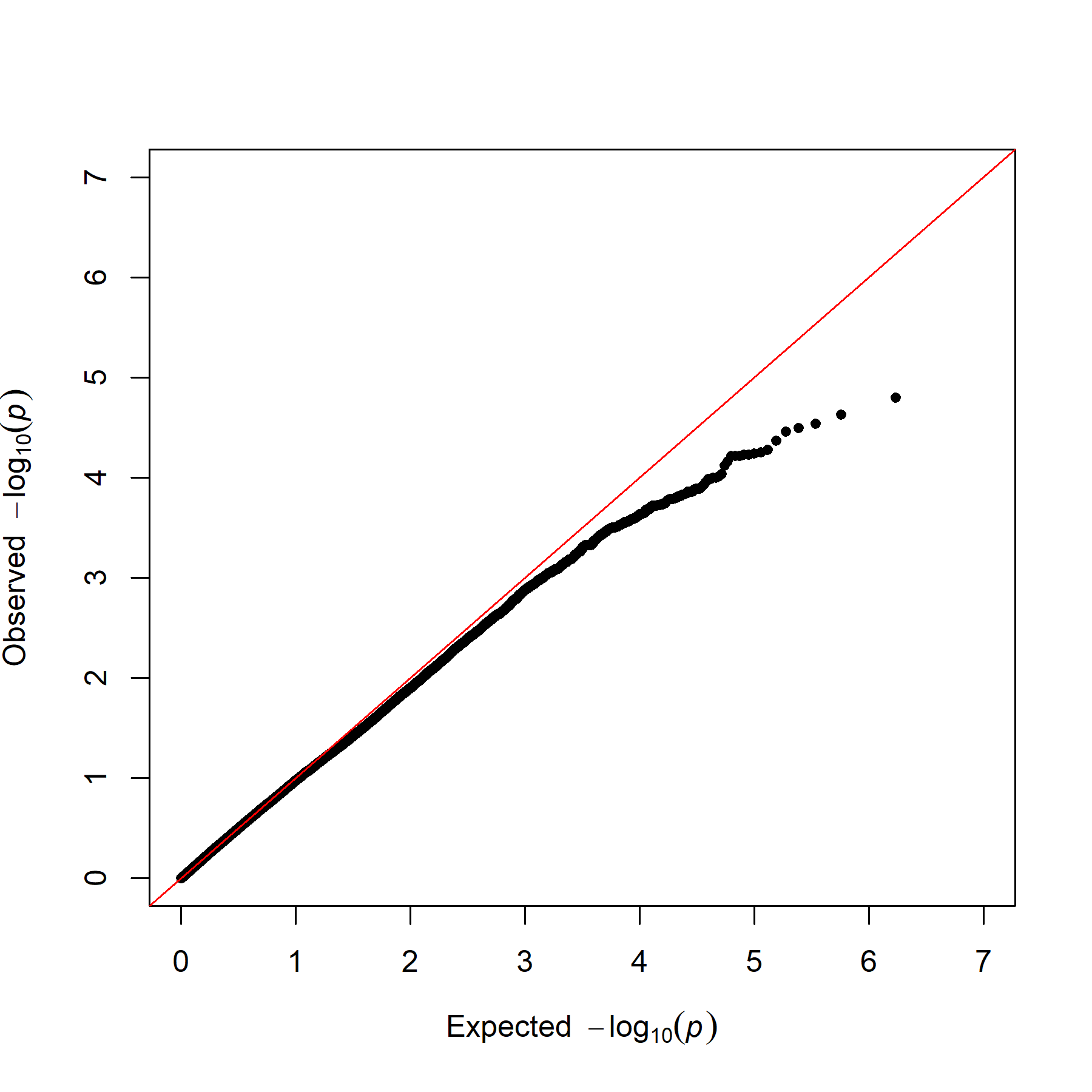


1. **MNPR (BLUP)**


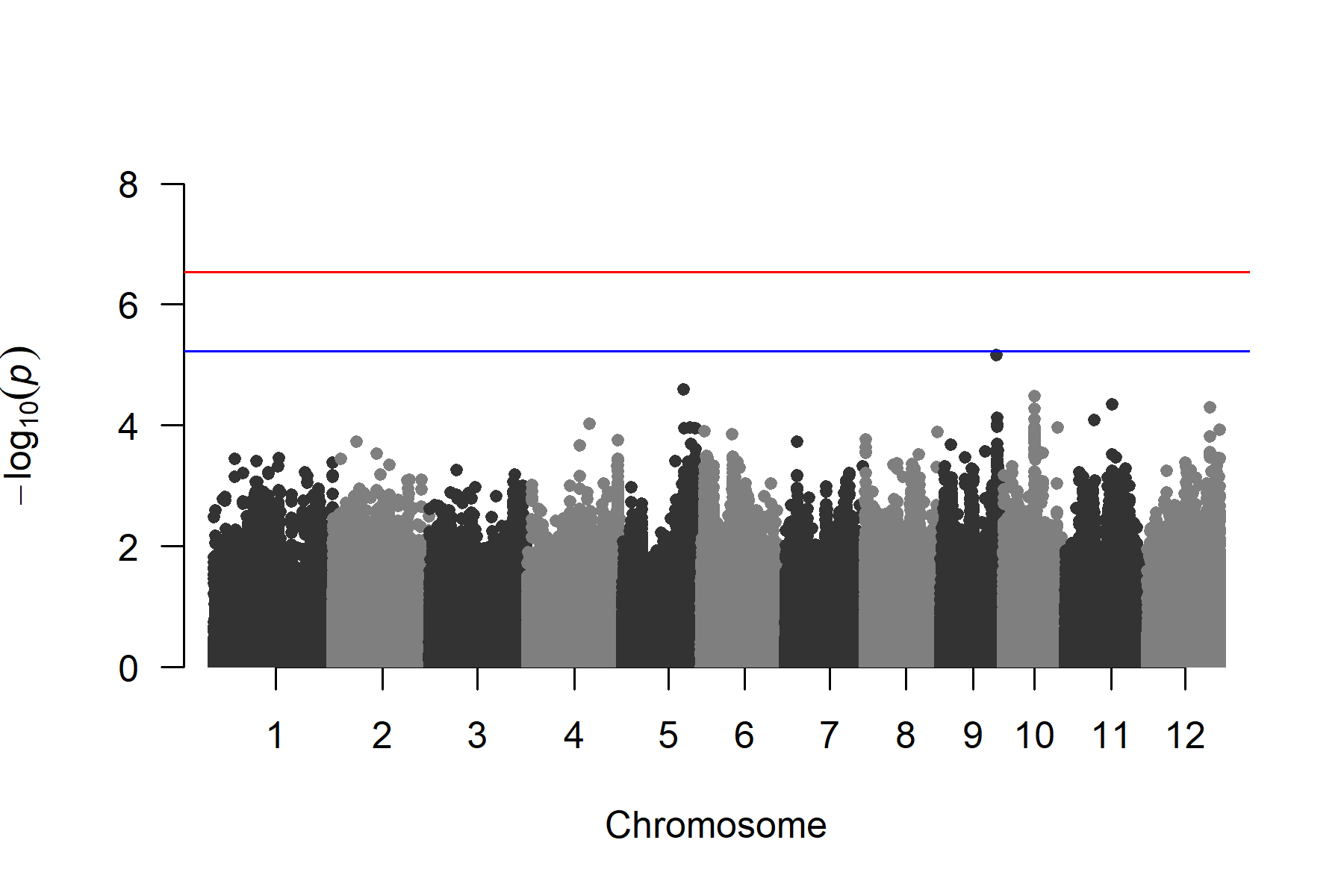

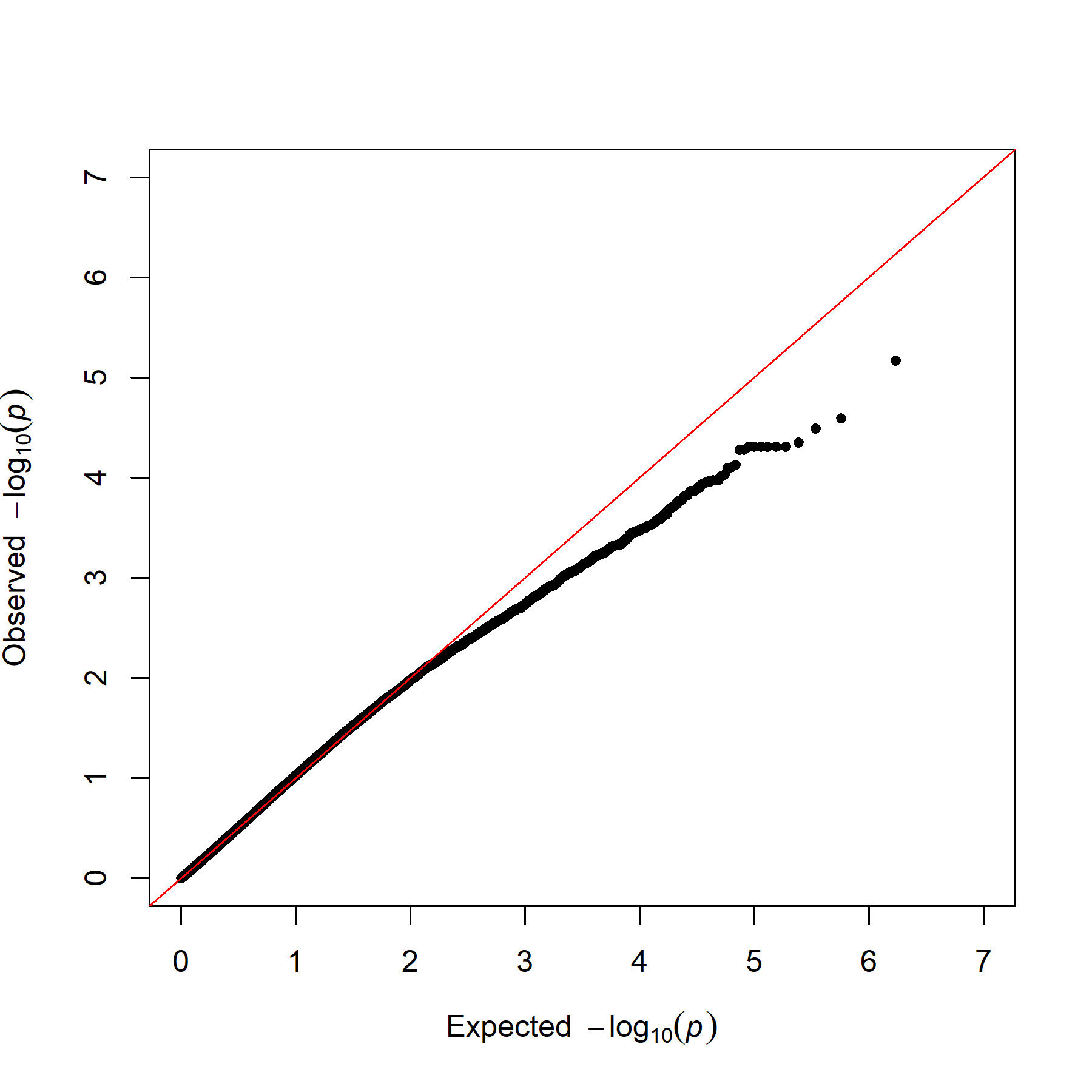


**Supplementary Figure 2**. Manhattan and Quantile-quantile (Q-Q) Plots showing suggestive SNP-trait association for (a) main culm panicle node number (MCPNN), (b-c) maximum node production rate (MNPR), and (d-e) degree days to heading (DDTH). The red line denotes the genome-wide significance threshold (P = 2.91 x 10^-7^), and the blue line denotes the suggestive threshold (P = 5.83 x 10^-6^).

1. **DDTH (2018)**


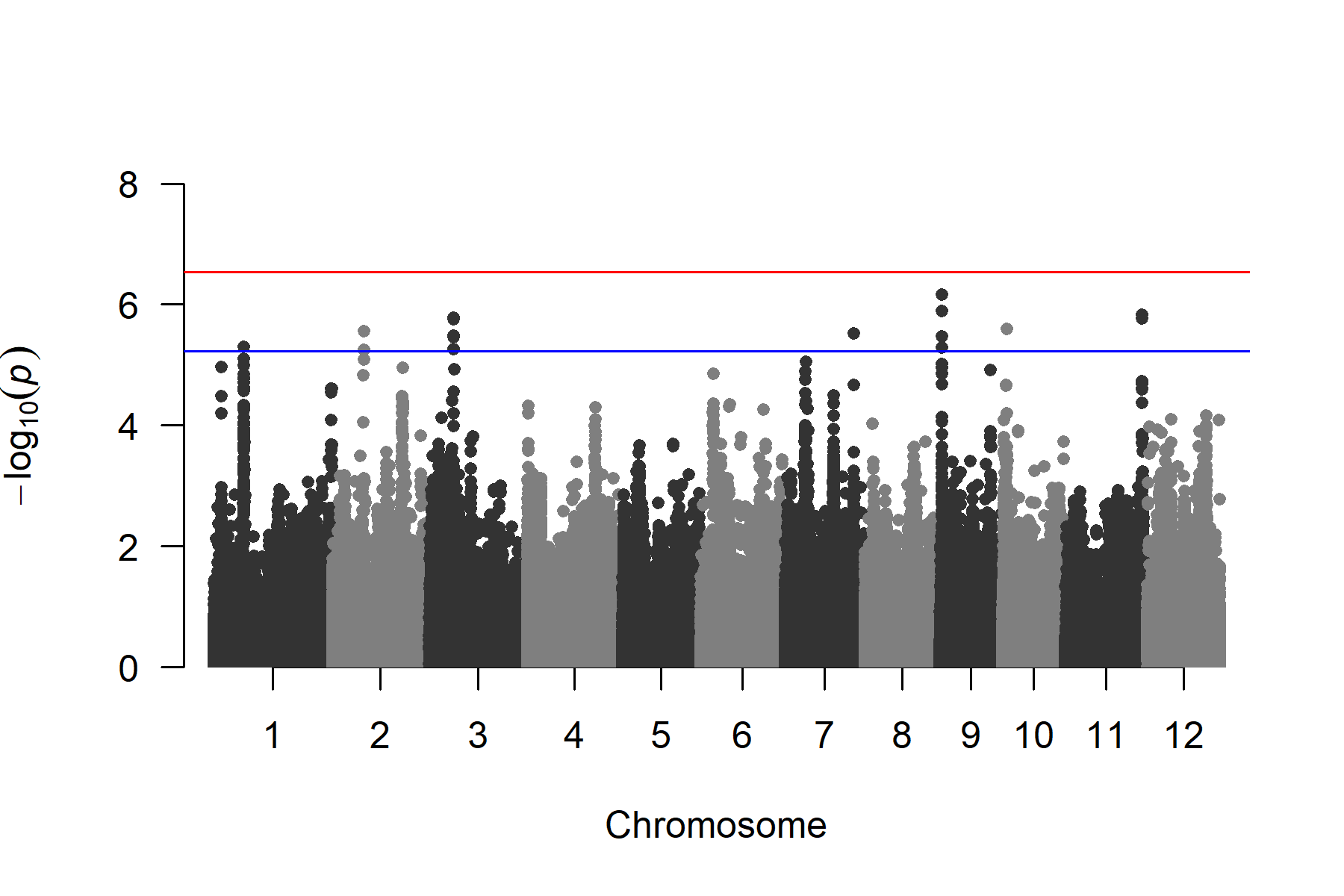

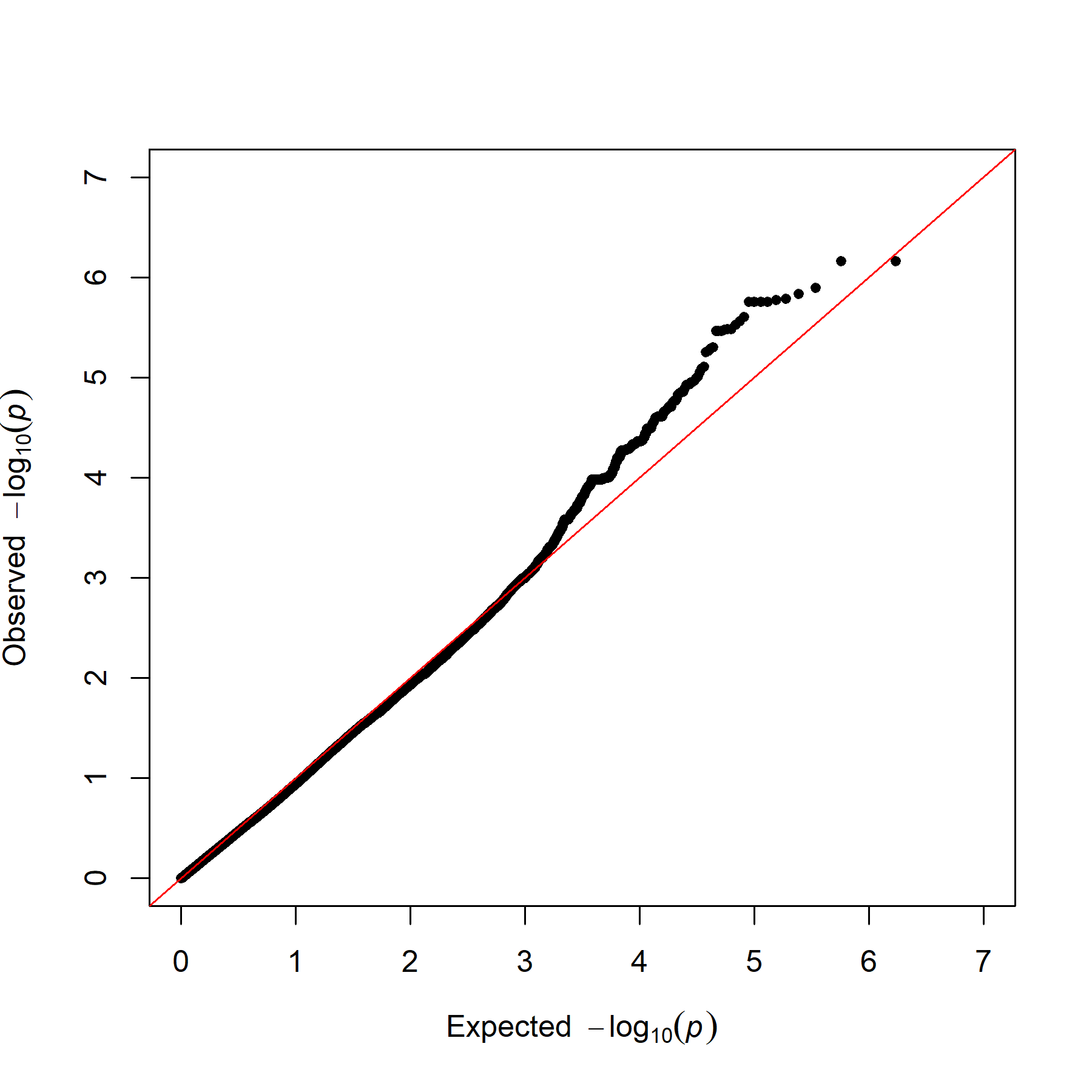


1. **DDTH (2019)**


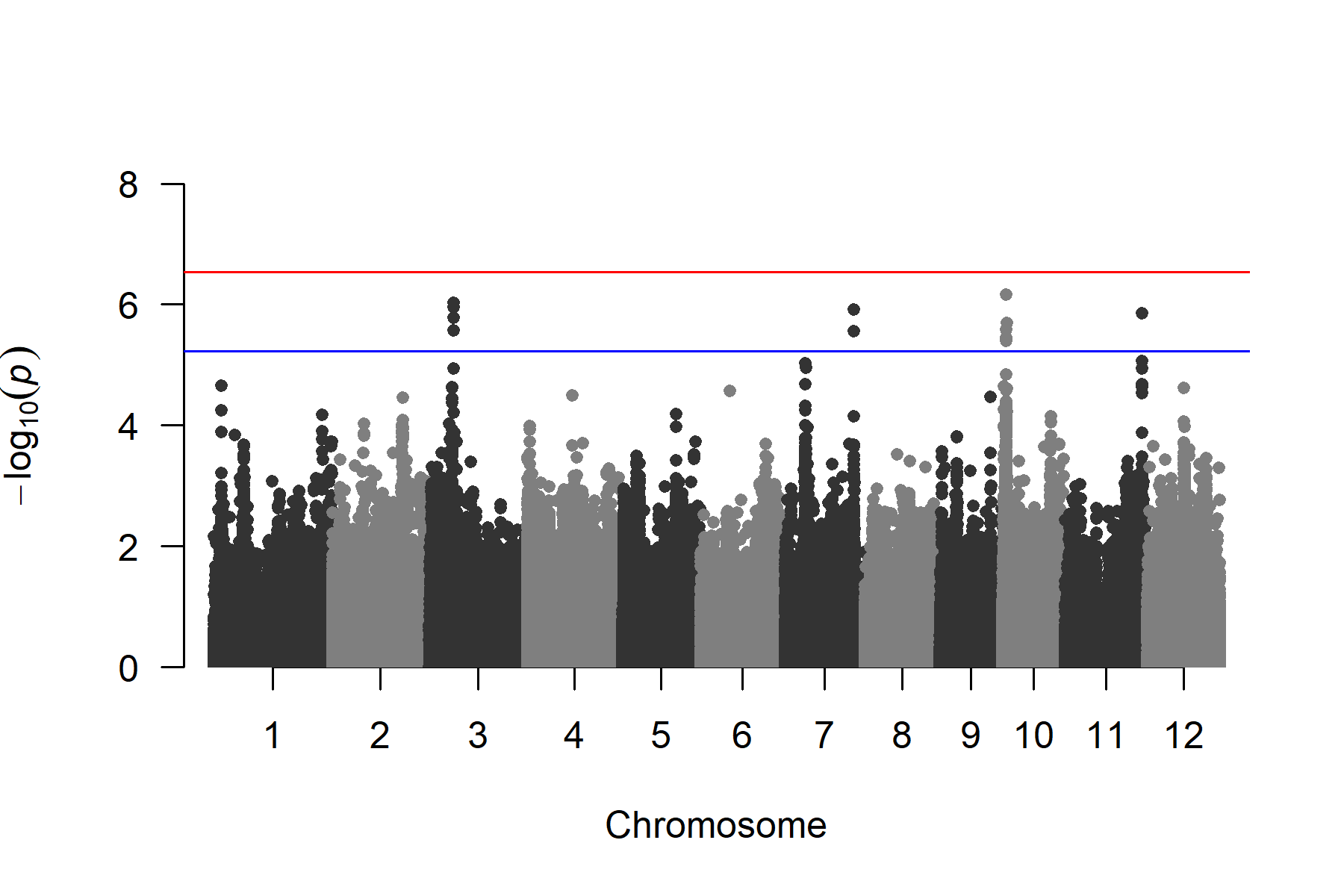

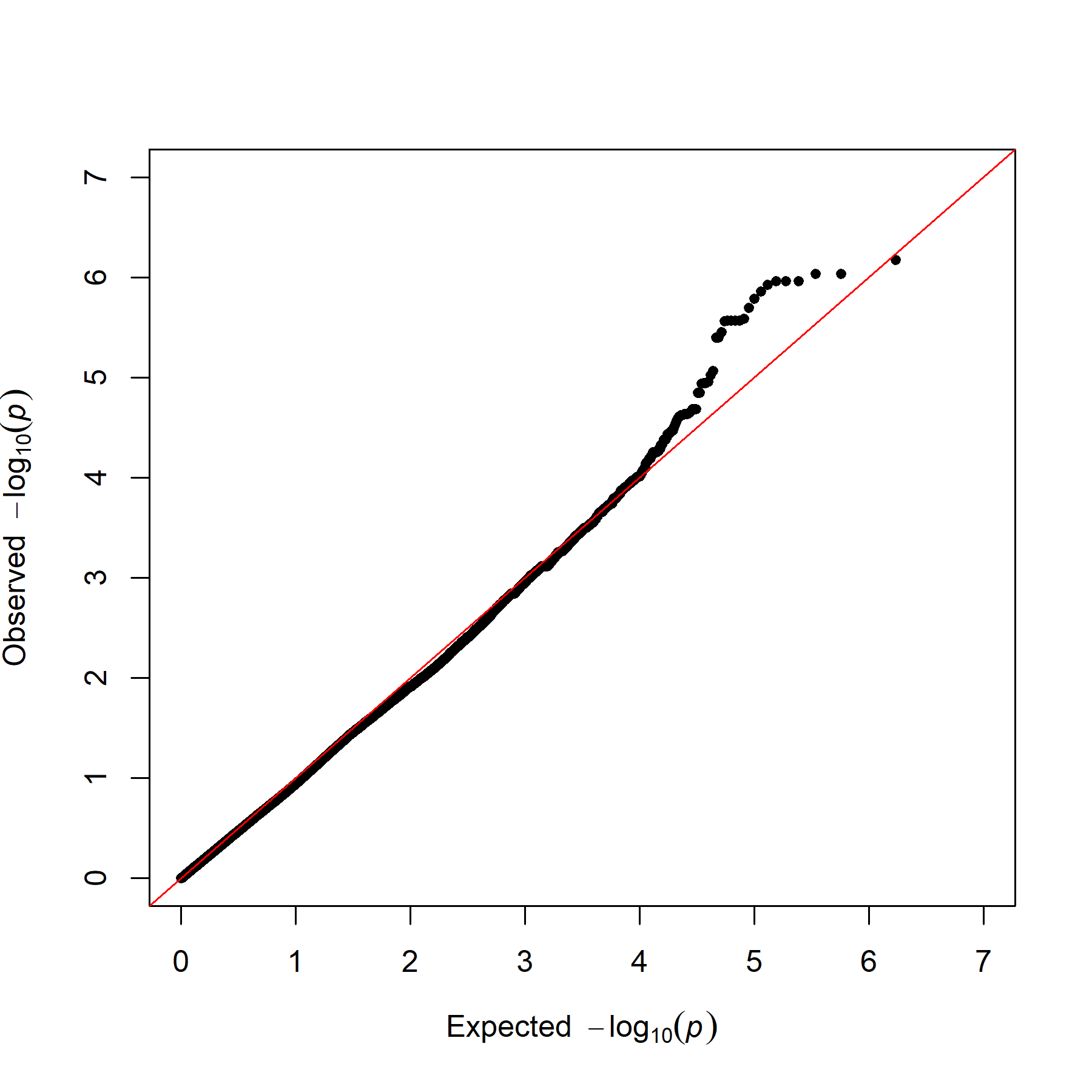


**Supplementary Figure 2.** (Continued)
