# Supplementary material for: Phenotypic variation and genome-wide association studies of main culm panicle node number, maximum node production rate, and degree-days to heading in rice
Source: BMC Genomics. 2022 May 23;23:390. doi: 10.1186/s12864-022-08629-y (PMC9125873; doi:10.1186/s12864-022-08629-y)
Supplement: Supplementary file 2 — Additional File 2. Supplementary Tables [file 12864_2022_8629_MOESM2_ESM.docx]

**Supplementary Table 1.** List of rice accessions used in the genome-wide association studies (GWAS) at Texas A&M AgriLife Research at Beaumont in 2018 and 2019.

|  | **Name** | **Country of Origin** | **Subgroup** |
| --- | --- | --- | --- |
| 1 | 119A/170R (Hybrid) | United States | Admixed |
| 2 | 119A/173R (Hybrid) | United States | Admixed |
| 3 | 119B | United States | Admixed |
| 4 | 152R | United States | Admixed |
| 5 | 153R | United States | Admixed |
| 6 | 163R | United States | Admixed |
| 7 | 164R | United States | Admixed |
| 8 | 170R | United States | Admixed |
| 9 | 171R | United States | Admixed |
| 10 | 172R | United States | Admixed |
| 11 | 173R | United States | Admixed |
| 12 | 329A/170R (Hybrid) | United States | Admixed |
| 13 | 329A/173R (Hybrid) | United States | Admixed |
| 14 | 329B | United States | Indica |
| 15 | 339A/170R (Hybrid) | United States | Admixed |
| 16 | 339A/173R (Hybrid) | United States | Admixed |
| 17 | 339B | United States | Admixed |
| 18 | 435A/170R (Hybrid) | United States | Admixed |
| 19 | 435A/173R (Hybrid) | United States | Admixed |
| 20 | 435B | United States | Admixed |
| 21 | 56-122-23 | Thailand | Temperate Japonica |
| 22 | 89-Y-235 | United States | Temperate Japonica |
| 23 | A 152 | Bangladesh | Admixed |
| 24 | Agostano | Italy | Temperate Japonica |
| 25 | Ai Yeh Lu | China | Temperate Japonica |
| 26 | Antonio | United States | Tropical Japonica |
| 27 | Ardito | Italy | Temperate Japonica |
| 28 | B6616A4-22-Bk-5-4 | United States | Tropical Japonica |
| 29 | Baber | India | Temperate Japonica |
| 30 | Bamoa A75 | Mexico | Indica |
| 31 | Bhim Dhan | Nepal | Admixed |
| 32 | Biser 1 | Bulgaria | Temperate Japonica |
| 33 | Blue Rose Supreme | United States | Admixed |
| 34 | Blue Stick | Fiji | Temperate Japonica |
| 35 | Bombilla | Spain | Temperate Japonica |
| 36 | Bombon | Spain | Temperate Japonica |
| 37 | Botika S/R | Zaire | Tropical Japonica |
| 38 | British Honduras Creole | Belize | Tropical Japonica |
| 39 | Bul Zo | Republic of Korea | Temperate Japonica |
| 40 | Buphopa | Myanmar | Admixed |
| 41 | C 5560 | Thailand | Admixed |
| 42 | C 8429 | Papua New Guinea | Tropical Japonica |
| 43 | C4-63 | Philippines | Indica |
| 44 | C57-5043 | United States | Tropical Japonica |
| 45 | Calmochi-101 | United States | Temperate Japonica |
| 46 | Camponi SML | Suriname | Admixed |
| 47 | Celiaj | Azerbaijan | Temperate Japonica |
| 48 | Cenit | Argentina | Tropical Japonica |
| 49 | Chacareiro Uruguay | Uruguay | Temperate Japonica |
| 50 | Cheniere | United States | Tropical Japonica |
| 51 | Chia Nung Yu 242 | Taiwan | Indica |
| 52 | Chunjiangzao No. 1 | China | Temperate Japonica |
| 53 | Cocodrie | United States | Tropical Japonica |
| 54 | Colorado | United States | Tropical Japonica |
| 55 | Coppocina | Bulgaria | Tropical Japonica |
| 56 | Criollo Chivacoa 2 | Venezuela | Tropical Japonica |
| 57 | Csornuj | Hungary | Temperate Japonica |
| 58 | Cybonnet | United States | Tropical Japonica |
| 59 | Cypress | United States | Tropical Japonica |
| 60 | Darmali | Nepal | Admixed |
| 61 | Della 2 | United States | Tropical Japonica |
| 62 | Diamond | United States | Tropical Japonica |
| 63 | Early | United States | Admixed |
| 64 | Early Colusa | United States | Temperate Japonica |
| 65 | Early Wataribune | Japan | Temperate Japonica |
| 66 | Edith | United States | Tropical Japonica |
| 67 | Edomen Scented | Japan | Temperate Japonica |
| 68 | Egyptian Wild Type | Turkey | Temperate Japonica |
| 69 | El Paso L-144 | Uruguay | Indica |
| 70 | Erythroceros Hokkaido | Poland | Temperate Japonica |
| 71 | Ginmasari | Japan | Temperate Japonica |
| 72 | Gogo Lempuk | Indonesia | Tropical Japonica |
| 73 | GPNO 1106 | Guatemala | Tropical Japonica |
| 74 | Guatemala 1021 | Guatemala | Tropical Japonica |
| 75 | H57-3-1 | Argentina | Temperate Japonica |
| 76 | Habiganj Boro 6 | Bangladesh | Admixed |
| 77 | Haginomae Mochi | Japan | Temperate Japonica |
| 78 | Hatsunishiki | Japan | Temperate Japonica |
| 79 | HB-6-2 | Hungary | Temperate Japonica |
| 80 | IAC 25 | Brazil | Tropical Japonica |
| 81 | IR 1321-12 | Philippines | Indica |
| 82 | IR24 | Philippines | Indica |
| 83 | IR-44595 | Nepal | Indica |
| 84 | IR64 | Philippines | Indica |
| 85 | IR64-Sub1 | Philippines | Indica |
| 86 | IR72 | Philippines | Indica |
| 87 | IRAT 44 | Burkina Faso | Tropical Japonica |
| 88 | IRAT 177 | French Guiana | Tropical Japonica |
| 89 | Italica Carolina | Poland | Temperate Japonica |
| 90 | J.P. 5 | Australia | Temperate Japonica |
| 91 | Jefferson | United States | Tropical Japonica |
| 92 | Jouiku 393G | Japan | Temperate Japonica |
| 93 | Kamenoo | Japan | Temperate Japonica |
| 94 | Karabaschak | Bulgaria | Temperate Japonica |
| 95 | Karang Serang | Indonesia | Tropical Japonica |
| 96 | Katy | United States | Tropical Japonica |
| 97 | Kaukkyi Ani | Myanmar | Admixed |
| 98 | Kaybonnet | United States | Tropical Japonica |
| 99 | Khao Luang | Laos | Admixed |
| 100 | Khao Phoi | Laos | Admixed |
| 101 | Kiuki No. 46 | Japan | Temperate Japonica |
| 102 | Koshihikari | Japan | Temperate Japonica |
| 103 | Krasnodarskij 3352 | Russian Federation | Temperate Japonica |
| 104 | Krasnodarskij 424 | Russian Federation | Temperate Japonica |
| 105 | L-201 | United States | Tropical Japonica |
| 106 | L-202 | United States | Tropical Japonica |
| 107 | L-203 | United States | Tropical Japonica |
| 108 | LAC 23 | Liberia | Tropical Japonica |
| 109 | Lady Wright Seln | United States | Tropical Japonica |
| 110 | LaGrue | United States | Tropical Japonica |
| 111 | LaKast | United States | Tropical Japonica |
| 112 | Lacassine | United States | Tropical Japonica |
| 113 | Leah | United States | Tropical Japonica |
| 114 | Lebonnet | United States | Tropical Japonica |
| 115 | Lemont | United States | Tropical Japonica |
| 116 | LGRU 2 | United States | Tropical Japonica |
| 117 | Ligerito | Colombia | Tropical Japonica |
| 118 | Lua Chua Chan | Vietnam | Tropical Japonica |
| 119 | Luk Takhar | Afghanistan | Temperate Japonica |
| 120 | Lusitano | Portugal | Temperate Japonica |
| 121 | M-201 | United States | Temperate Japonica |
| 122 | M-202 | United States | Admixed |
| 123 | M-203 | United States | Temperate Japonica |
| 124 | M-204 | United States | Temperate Japonica |
| 125 | M-401 | United States | Temperate Japonica |
| 126 | Mars | United States | Tropical Japonica |
| 127 | Mercury | United States | Admixed |
| 128 | Minghui 63 | China | Indica |
| 129 | Mojito Colorado | Bolivia | Tropical Japonica |
| 130 | Moroberekan | Guinea | Tropical Japonica |
| 131 | N22 | India | Aus |
| 132 | Nanton No. 131 | Taiwan | Admixed |
| 133 | Newbonnet | United States | Tropical Japonica |
| 134 | Nipponbare | Japan | Temperate Japonica |
| 135 | Niwahutaw Mochi | Japan | Temperate Japonica |
| 136 | Nortai | United States | Admixed |
| 137 | Nova | United States | Admixed |
| 138 | Noventa Dias Blanco | Bolivia | Tropical Japonica |
| 139 | NSF-TV 107 | Bangladesh | Tropical Japonica |
| 140 | Oryzica Llanos 4 | Colombia | Indica |
| 141 | Oryzica Llanos 5 | Colombia | Indica |
| 142 | OS 6 (WC 10296) | Nigeria | Tropical Japonica |
| 143 | Padi Pohon Batu | Malaysia | Tropical Japonica |
| 144 | Palmyra | United States | Tropical Japonica |
| 145 | Panda | Italy | Admixed |
| 146 | Pergonil 15 | Portugal | Temperate Japonica |
| 147 | Presidio | United States | Tropical Japonica |
| 148 | Quinimpol | Philippines | Tropical Japonica |
| 149 | R 101 | Zaire | Tropical Japonica |
| 150 | R 67 | Senegal | Tropical Japonica |
| 151 | R 75 | Senegal | Tropical Japonica |
| 152 | Ragasu | Taiwan | Admixed |
| 153 | RD 218 | Dominican Republic | Admixed |
| 154 | Rex | United States | Tropical Japonica |
| 155 | Rico 1 | United States | Tropical Japonica |
| 156 | Romanica | Hungary | Temperate Japonica |
| 157 | Romeno | Portugal | Temperate Japonica |
| 158 | Romeo | Italy | Temperate Japonica |
| 159 | Rondo | United States | Indica |
| 160 | ROY J | United States | Tropical Japonica |
| 161 | RT 1031-69 | Zaire | Tropical Japonica |
| 162 | RU-903147 | United States | Japonica |
| 163 | RU-1003098 | United States | Tropical Japonica |
| 164 | RU-1003123 | United States | Tropical Japonica |
| 165 | RU-1303181 | United States | Tropical Japonica |
| 166 | RU-1303184 | United States | Japonica |
| 167 | RU-1403138 | United States | Tropical Japonica |
| 168 | RU-1403141 | United States | Tropical Japonica |
| 169 | RU-1403166 | United States | Tropical Japonica |
| 170 | RU-1503110 | United States | Tropical Japonica |
| 171 | RU-1503147 | United States | Tropical Japonica |
| 172 | RU-1603126 | United States | Tropical Japonica |
| 173 | RU-1603150 | United States | Tropical Japonica |
| 174 | Saber | United States | Tropical Japonica |
| 175 | Sabine | United States | Tropical Japonica |
| 176 | Sathi | Pakistan | Aus |
| 177 | Saturn | United States | Tropical Japonica |
| 178 | Secano do Brazil | El Salvador | Tropical Japonica |
| 179 | Sel. No. 388 | Uruguay | Admixed |
| 180 | Shimizu Mochi | Japan | Temperate Japonica |
| 181 | Shinriki | Japan | Temperate Japonica |
| 182 | Shirogane | Japan | Temperate Japonica |
| 183 | Short Grain | Thailand | Indica |
| 184 | Simpor | Brunei | Tropical Japonica |
| 185 | Sipirasikkam | Indonesia | Tropical Japonica |
| 186 | Sml Kapuri | Suriname | Temperate Japonica |
| 187 | Somewake | Japan | Temperate Japonica |
| 188 | Sri Malaysia Dua | Malaysia | Temperate Japonica |
| 189 | Sultani | Egypt | Tropical Japonica |
| 190 | Sung Liao 2 | China | Temperate Japonica |
| 191 | Suweon | Republic of Korea | Temperate Japonica |
| 192 | Ta Hung Ku | China | Temperate Japonica |
| 193 | Taichu Mochi 59 | Taiwan | Tropical Japonica |
| 194 | Tainan-Iku No. 512 | Taiwan | Temperate Japonica |
| 195 | Taipei 309 | Taiwan | Temperate Japonica |
| 196 | Takao No. 25 | Taiwan | Unknown |
| 197 | Takao-Iku No. 44 | Taiwan | Unknown |
| 198 | Takao-Iku No. 8 | Taiwan | Unknown |
| 199 | Tamanishiki | Japan | Temperate Japonica |
| 200 | Terso | United States | Temperate Japonica |
| 201 | Thad | United States | Tropical Japonica |
| 202 | Tia Bura | Indonesia | Tropical Japonica |
| 203 | Titan | United States | Temperate Japonica |
| 204 | Tokyo Shino Mochi | Japan | Admixed |
| 205 | Tox 782-20-1 | Nigeria | Tropical Japonica |
| 206 | Upland | Ponape Island | Tropical Japonica |
| 207 | UZ ROSZ M38 | Uzbekistan | Temperate Japonica |
| 208 | Vary Tarva Osla | Portugal | Temperate Japonica |
| 209 | Very Early M9 | United States | Temperate Japonica |
| 210 | WAB462-10-3-1 | Cote D'Ivoire | Tropical Japonica |
| 211 | WC 2811 | Micronesia | Tropical Japonica |
| 212 | WC 3397 | Jamaica | Tropical Japonica |
| 213 | WC 3532 | Peru | Tropical Japonica |
| 214 | WC 4443 | Bolivia | Tropical Japonica |
| 215 | WC 6 | China | Temperate Japonica |
| 216 | Wells | United States | Tropical Japonica |
| 217 | WIR 3039 | Tajikistan | Temperate Japonica |
| 218 | WW 8/2290 | Netherlands | Admixed |
| 219 | Yong Chal Byo | Republic of Korea | Temperate Japonica |
| 220 | Zhenshan 2 | China | Indica |

**Supplementary Table 2. List of gene models located within 100 kilobase pairs of SNPs significantly associated with main culm panicle node number, maximum node production rate and degree-days to heading in rice.**

| **Trait** | **Gene Model (IRGSP Build 5)** | **Chromosome** | **Start** | **End** | **Direction** | **Annotation** | **Significant SNP** | **Distance to Significant SNP (bp)** |
| --- | --- | --- | --- | --- | --- | --- | --- | --- |
| MCPNN | Os02g0304500 | 2 | 11,895,000 | 11,898,798 | - | Similar to transposon protein | S02_11971745 | -72,947 |
| MCPNN | Os02g0304701 | 2 | 11,903,862 | 11,904,909 | + | Hypothetical conserved gene | S02_11971745 | -66,836 |
| MCPNN | Os02g0304800 | 2 | 11,905,332 | 11,908,827 | + | Hypothetical conserved gene | S02_11971745 | -62,918 |
| MCPNN | Os02g0304900 | 2 | 11,911,302 | 11,914,857 | - | Drought induced 19 family protein | S02_11971745 | -56,888 |
| MCPNN | Os02g0305600 | 2 | 11,964,314 | 11,970,762 | - | Spectrin repeat containing protein | S02_11971745 | -983 |
|  |  |  |  |  |  |  | S02_12030176 | -59,414 |
|  |  |  |  |  |  |  | S02_12032235 | -61,473 |
| MCPNN | Os02g0305800 | 2 | 11,973,414 | 11,978,894 | + | EKC/KEOPS complex, subunit Pcc1 domain containing protein | S02_11971745 | 1,669 |
|  |  |  |  |  |  |  | S02_12030176 | -51,282 |
|  |  |  |  |  |  |  | S02_12032235 | -53,341 |
| MCPNN | Os02g0305700 | 2 | 11,990,724 | 12,005,210 | + | Armadillo-type fold domain containing protein | S02_11971745 | 18,979 |
|  |  |  |  |  |  |  | S02_12030176 | -24,966 |
|  |  |  |  |  |  |  | S02_12032235 | -27,025 |
| MCPNN | Os02g0305950 | 2 | 11,997,301 | 11,997,732 | - | Similar to calmodulin binding protein; Auxin-responsive SAUR protein | S02_11971745 | 25,556 |
|  |  |  |  |  |  |  | S02_12030176 | -32,444 |
|  |  |  |  |  |  |  | S02_12032235 | -34,503 |
| MCPNN | Os02g0306100 | 2 | 12,000,127 | 12,002,515 | + | Similar to cation-cation antiporter; vesicle transport protein, Use1 | S02_11971745 | 28,382 |
|  |  |  |  |  |  |  | S02_12030176 | -27,661 |
|  |  |  |  |  |  |  | S02_12032235 | -29,720 |
| MCPNN | Os02g0306401 | 2 | 12,026,212 | 12,031,621 | - | Similar to Nicotianamine aminotransferase A | S02_11971745 | 54,467 |
|  |  |  |  |  |  |  | S02_12030176 | 0 |
|  |  |  |  |  |  |  | S02_12032235 | -614 |
| MCPNN | Os02g0306600 | 2 | 12,038,030 | 12,038,415 | - | Non-protein coding transcript | S02_11971745 | 66,285 |
|  |  |  |  |  |  |  | S02_12030176 | 7,854 |
|  |  |  |  |  |  |  | S02_12032235 | 5,975 |
| MCPNN | Os02g0306701 | 2 | 12,040,328 | 12,044,433 | - | Hypothetical conserved gene | S02_11971745 | 68,583 |
|  |  |  |  |  |  |  | S02_12030176 | 10,152 |
|  |  |  |  |  |  |  | S02_12032235 | 8,093 |
| MCPNN | Os02g0306900 | 2 | 12,048,030 | 12,052,440 | + | Similar to DnaJ-like protein | S02_11971745 | 76,285 |
|  |  |  |  |  |  |  | S02_12030176 | 17,854 |
|  |  |  |  |  |  |  | S02_12032235 | 15,795 |
| MCPNN | Os02g0306801 | 2 | 12,048,124 | 12,049,453 | - | Hypothetical gene | S02_11971745 | 76,379 |
|  |  |  |  |  |  |  | S02_12030176 | 17,948 |
|  |  |  |  |  |  |  | S02_12032235 | 15,889 |
| MCPNN | Os02g0307000 | 2 | 12,052,915 | 12,063,637 | - | Guanylate-binding protein family protein | S02_11971745 | 81,170 |
|  |  |  |  |  |  |  | S02_12030176 | 22,739 |
|  |  |  |  |  |  |  | S02_12032235 | 20,680 |
| MCPNN | Os02g0307050 | 2 | 12,060,087 | 12,06,3136 | + | Hypothetical gene | S02_11971745 | 88,342 |
|  |  |  |  |  |  |  | S02_12030176 | 29,911 |
|  |  |  |  |  |  |  | S02_12032235 | 27,852 |
| MCPNN | Os02g0307200 | 2 | 12,083,767 | 12,086,597 | + | Similar to transducin family protein/ WD-40 repeat family protein | S02_12030176 | 53,591 |
|  |  |  |  |  |  |  | S02_12032235 | 51,532 |
| MCPNN | Os02g0307300 | 2 | 12,086,454 | 12,088,376 | - | Hypothetical conserved gene | S02_12030176 | 56,278 |
|  |  |  |  |  |  |  | S02_12032235 | 54,219 |
| MCPNN | Os02g0307800 | 2 | 12,115,013 | 12,118,345 | + | Protein of unknown function DUF3082 domain containing protein | S02_12030176 | 84,837 |
|  |  |  |  |  |  |  | S02_12032235 | 82,778 |
| MNPR | Os06g0134800 | 6 | 1,871,647 | 1,874,894 | - | Folate-binding, YgfZ domain containing protein | S06_1968653 | -93,759 |
|  |  |  |  |  |  |  | S06_1968680 | -93,786 |
|  |  |  |  |  |  |  | S06_1968681 | -93,787 |
|  |  |  |  |  |  |  | S06_1970442 | -95,548 |
|  |  |  |  |  |  |  | S06_1970597 | -95,703 |
|  |  |  |  |  |  |  | S06_1970602 | -95,708 |
| MNPR | Os06g0134900 | 6 | 1,875,621 | 1,879,253 | - | Conserved hypothetical protein | S06_1968653 | -89,400 |
|  |  |  |  |  |  |  | S06_1968680 | -89,427 |
|  |  |  |  |  |  |  | S06_1968681 | -89,428 |
|  |  |  |  |  |  |  | S06_1970442 | -91,189 |
|  |  |  |  |  |  |  | S06_1970597 | -91,344 |
|  |  |  |  |  |  |  | S06_1970602 | -91,349 |
| MNPR | Os06g0135000 | 6 | 1,879,538 | 1,889,910 | + | Similar to hypersensitive-induced reaction protein 4 | S06_1968653 | -78,743 |
|  |  |  |  |  |  |  | S06_1968680 | -78,770 |
|  |  |  |  |  |  |  | S06_1968681 | -78,771 |
|  |  |  |  |  |  |  | S06_1970442 | -80,532 |
|  |  |  |  |  |  |  | S06_1970597 | -80,687 |
|  |  |  |  |  |  |  | S06_1970602 | -80,692 |
| MNPR | Os06g0135460 | 6 | 1,892,930 | 1,893,328 | - | Similar to Lipoxygenase | S06_1968653 | -75,325 |
|  |  |  |  |  |  |  | S06_1968680 | -75,352 |
|  |  |  |  |  |  |  | S06_1968681 | -75,353 |
|  |  |  |  |  |  |  | S06_1970442 | -77,114 |
|  |  |  |  |  |  |  | S06_1970597 | -77,269 |
|  |  |  |  |  |  |  | S06_1970602 | -77,274 |
| MNPR | Os06g0135300 | 6 | 1,892,172 | 1,893,619 | + | Conserved hypothetical protein | S06_1968653 | -75,034 |
|  |  |  |  |  |  |  | S06_1968680 | -75,061 |
|  |  |  |  |  |  |  | S06_1968681 | -75,062 |
|  |  |  |  |  |  |  | S06_1970442 | -76,823 |
|  |  |  |  |  |  |  | S06_1970597 | -76,978 |
|  |  |  |  |  |  |  | S06_1970602 | -76,983 |
| MNPR | Os06g0135900 | 6 | 1,899,869 | 1,907,947 | - | Similar to Sec1p-like protein 2 (Fragment) | S06_1968653 | -60,706 |
|  |  |  |  |  |  |  | S06_1968680 | -60,733 |
|  |  |  |  |  |  |  | S06_1968681 | -60,734 |
|  |  |  |  |  |  |  | S06_1970442 | -62,495 |
|  |  |  |  |  |  |  | S06_1970597 | -62,650 |
|  |  |  |  |  |  |  | S06_1970602 | -62,655 |
| MNPR | Os06g0136000 | 6 | 1,908,696 | 1,912,031 | - | Similar to Hypersensitive-induced reaction protein 4 | S06_1968653 | -56,622 |
|  |  |  |  |  |  |  | S06_1968680 | -56,649 |
|  |  |  |  |  |  |  | S06_1968681 | -56,650 |
|  |  |  |  |  |  |  | S06_1970442 | -58,411 |
|  |  |  |  |  |  |  | S06_1970597 | -58,566 |
|  |  |  |  |  |  |  | S06_1970602 | -58,571 |
| MNPR | Os06g0136100 | 6 | 1,913,193 | 1,917,008 | - | Conserved hypothetical protein | S06_1968653 | -51,645 |
|  |  |  |  |  |  |  | S06_1968680 | -51,672 |
|  |  |  |  |  |  |  | S06_1968681 | -51,673 |
|  |  |  |  |  |  |  | S06_1970442 | -53,434 |
|  |  |  |  |  |  |  | S06_1970597 | -53,589 |
|  |  |  |  |  |  |  | S06_1970602 | -53,594 |
| MNPR | Os06g0136201 | 6 | 1,920,203 | 1,928,312 | - | Hypothetical gene | S06_1968653 | -40,341 |
|  |  |  |  |  |  |  | S06_1968680 | -40,368 |
|  |  |  |  |  |  |  | S06_1968681 | -40,369 |
|  |  |  |  |  |  |  | S06_1970442 | -42,130 |
|  |  |  |  |  |  |  | S06_1970597 | -42,285 |
|  |  |  |  |  |  |  | S06_1970602 | -42,290 |
| MNPR | Os06g0136300 | 6 | 1,928,312 | 1,922,594 | + | Similar to OSIGBa0142C11.2 protein | S06_1968653 | -46,059 |
|  |  |  |  |  |  |  | S06_1968680 | -46,086 |
|  |  |  |  |  |  |  | S06_1968681 | -46,087 |
|  |  |  |  |  |  |  | S06_1970442 | -47,848 |
|  |  |  |  |  |  |  | S06_1970597 | -48,003 |
|  |  |  |  |  |  |  | S06_1970602 | -48,008 |
| MNPR | Os06g0136500 | 6 | 1,926,257 | 1,929,768 | + | Cornichon family protein | S06_1968653 | -38,885 |
|  |  |  |  |  |  |  | S06_1968680 | -38,912 |
|  |  |  |  |  |  |  | S06_1968681 | -38,913 |
|  |  |  |  |  |  |  | S06_1970442 | -40,674 |
|  |  |  |  |  |  |  | S06_1970597 | -40,829 |
|  |  |  |  |  |  |  | S06_1970602 | -40,834 |
| MNPR | Os06g0136600 | 6 | 1,930,306 | 1,935,377 | - | Similar to Enolase | S06_1968653 | -33,276 |
|  |  |  |  |  |  |  | S06_1968680 | -33,303 |
|  |  |  |  |  |  |  | S06_1968681 | -33,304 |
|  |  |  |  |  |  |  | S06_1970442 | -35,065 |
|  |  |  |  |  |  |  | S06_1970597 | -35,220 |
|  |  |  |  |  |  |  | S06_1970602 | -35,225 |
| MNPR | Os06g0136700 | 6 | 1,936,858 | 1,947,666 | + | Steroid nuclear receptor, ligand-binding domain containing protein | S06_1968653 | -20,987 |
|  |  |  |  |  |  |  | S06_1968680 | -21,014 |
|  |  |  |  |  |  |  | S06_1968681 | -21,015 |
|  |  |  |  |  |  |  | S06_1970442 | -22,776 |
|  |  |  |  |  |  |  | S06_1970597 | -22,931 |
|  |  |  |  |  |  |  | S06_1970602 | -22,936 |
| MNPR | Os06g0136800 | 6 | 1,948,465 | 1,952,285 | + | Peptidase S14, ClpP family protein | S06_1968653 | -16,368 |
|  |  |  |  |  |  |  | S06_1968680 | -16,395 |
|  |  |  |  |  |  |  | S06_1968681 | -16,396 |
|  |  |  |  |  |  |  | S06_1970442 | -18,157 |
|  |  |  |  |  |  |  | S06_1970597 | -18,312 |
|  |  |  |  |  |  |  | S06_1970602 | -18,317 |
| MNPR | Os06g0136900 | 6 | 1,961,307 | 1,962,581 | - | Nuclear-localized AT-hook DNA binding protein, Regulation of palea development, Control of floral organ number | S06_1968653 | -6,072 |
|  |  |  |  |  |  |  | S06_1968680 | -6,099 |
|  |  |  |  |  |  |  | S06_1968681 | -6,100 |
|  |  |  |  |  |  |  | S06_1970442 | -7,861 |
|  |  |  |  |  |  |  | S06_1970597 | -8,016 |
|  |  |  |  |  |  |  | S06_1970602 | -8,021 |
| MNPR | Os06g0137100 | 6 | 1,972,537 | 1,975,557 | - | Similar to predicted protein | S06_1968653 | 3,884 |
|  |  |  |  |  |  |  | S06_1968680 | 3,857 |
|  |  |  |  |  |  |  | S06_1968681 | 3,856 |
|  |  |  |  |  |  |  | S06_1970442 | 2,095 |
|  |  |  |  |  |  |  | S06_1970597 | 1,940 |
|  |  |  |  |  |  |  | S06_1970602 | 1,935 |
| MNPR | Os06g0137166 | 6 | 1,977,442 | 1,980,622 | + | Hypothetical gene | S06_1968653 | 8,789 |
|  |  |  |  |  |  |  | S06_1968680 | 8,762 |
|  |  |  |  |  |  |  | S06_1968681 | 8,761 |
|  |  |  |  |  |  |  | S06_1970442 | 7,000 |
|  |  |  |  |  |  |  | S06_1970597 | 6,845 |
|  |  |  |  |  |  |  | S06_1970602 | 6,840 |
| MNPR | Os06g0137300 | 6 | 1,989,030 | 1,992,642 | + | YEATS family protein | S06_1968653 | 20,377 |
|  |  |  |  |  |  |  | S06_1968680 | 20,350 |
|  |  |  |  |  |  |  | S06_1968681 | 20,349 |
|  |  |  |  |  |  |  | S06_1970442 | 18,588 |
|  |  |  |  |  |  |  | S06_1970597 | 18,433 |
|  |  |  |  |  |  |  | S06_1970602 | 18,428 |
| MNPR | Os06g0137400 | 6 | 1,999,211 | 1,999,570 | + | Hypothetical conserved gene; Auxin responsive SAUR protein | S06_1968653 | 30,558 |
|  |  |  |  |  |  |  | S06_1968680 | 30,531 |
|  |  |  |  |  |  |  | S06_1968681 | 30,530 |
|  |  |  |  |  |  |  | S06_1970442 | 28,769 |
|  |  |  |  |  |  |  | S06_1970597 | 28,614 |
|  |  |  |  |  |  |  | S06_1970602 | 28,609 |
| MNPR | Os06g0137500 | 6 | 1,999,873 | 2,008,069 | - | Brix domain containing protein | S06_1968653 | 31,220 |
|  |  |  |  |  |  |  | S06_1968680 | 31,193 |
|  |  |  |  |  |  |  | S06_1968681 | 31,192 |
|  |  |  |  |  |  |  | S06_1970442 | 29,431 |
|  |  |  |  |  |  |  | S06_1970597 | 29,276 |
|  |  |  |  |  |  |  | S06_1970602 | 29,271 |
| MNPR | Os06g0137600 | 6 | 2,008,997 | 2,014,660 | + | K homology-like, alpha/beta domain containing protein | S06_1968653 | 40,344 |
|  |  |  |  |  |  |  | S06_1968680 | 40,317 |
|  |  |  |  |  |  |  | S06_1968681 | 40,316 |
|  |  |  |  |  |  |  | S06_1970442 | 38,555 |
|  |  |  |  |  |  |  | S06_1970597 | 38,400 |
|  |  |  |  |  |  |  | S06_1970602 | 38,395 |
| MNPR | Os06g0137650 | 6 | 2,016,979 | 2,020,998 | - | UDP-glucuronosyl/UDP-glucosyltransferase domain containing protein | S06_1968653 | 48,326 |
|  |  |  |  |  |  |  | S06_1968680 | 48,299 |
|  |  |  |  |  |  |  | S06_1968681 | 48,298 |
|  |  |  |  |  |  |  | S06_1970442 | 46,537 |
|  |  |  |  |  |  |  | S06_1970597 | 46,382 |
|  |  |  |  |  |  |  | S06_1970602 | 46,377 |
| MNPR | Os06g0137700 | 6 | 2,016,779 | 2,019,680 | + | Similar to GDP-mannose 4,6 dehydratase 2 | S06_1968653 | 48,126 |
|  |  |  |  |  |  |  | S06_1968680 | 48,099 |
|  |  |  |  |  |  |  | S06_1968681 | 48,098 |
|  |  |  |  |  |  |  | S06_1970442 | 46,337 |
|  |  |  |  |  |  |  | S06_1970597 | 46,182 |
|  |  |  |  |  |  |  | S06_1970602 | 46,177 |
| MNPR | Os06g0138000 | 6 | 2,026,184 | 2,026,944 | + | Similar to IMB1 | S06_1968653 | 57,531 |
|  |  |  |  |  |  |  | S06_1968680 | 57,504 |
|  |  |  |  |  |  |  | S06_1968681 | 57,503 |
|  |  |  |  |  |  |  | S06_1970442 | 55,742 |
|  |  |  |  |  |  |  | S06_1970597 | 55,587 |
|  |  |  |  |  |  |  | S06_1970602 | 55,582 |
| MNPR | Os06g0138100 | 6 | 2,027,123 | 2,029,212 | - | Methionine sulphoxide reductase A domain containing protein | S06_1968653 | 58,470 |
|  |  |  |  |  |  |  | S06_1968680 | 58,443 |
|  |  |  |  |  |  |  | S06_1968681 | 58,442 |
|  |  |  |  |  |  |  | S06_1970442 | 56,681 |
|  |  |  |  |  |  |  | S06_1970597 | 56,526 |
|  |  |  |  |  |  |  | S06_1970602 | 56,521 |
| MNPR | Os06g0138200 | 6 | 2,029,472 | 2,033,740 | + | 2OG-Fe(II) oxygenase domain containing protein | S06_1968653 | 60,819 |
|  |  |  |  |  |  |  | S06_1968680 | 60,792 |
|  |  |  |  |  |  |  | S06_1968681 | 60,791 |
|  |  |  |  |  |  |  | S06_1970442 | 59,030 |
|  |  |  |  |  |  |  | S06_1970597 | 58,875 |
|  |  |  |  |  |  |  | S06_1970602 | 58,870 |
| MNPR | Os06g0138400 | 6 | 2,035,652 | 2,036,779 | + | Hypothetical conserved gene | S06_1968653 | 66,999 |
|  |  |  |  |  |  |  | S06_1968680 | 66,972 |
|  |  |  |  |  |  |  | S06_1968681 | 66,971 |
|  |  |  |  |  |  |  | S06_1970442 | 65,210 |
|  |  |  |  |  |  |  | S06_1970597 | 65,055 |
|  |  |  |  |  |  |  | S06_1970602 | 65,050 |
| MNPR | Os06g0138600 | 6 | 2,038,737 | 2,040,428 | - | Protein of unknown function DUF248, methyltransferase putative domain containing protein | S06_1968653 | 70,084 |
|  |  |  |  |  |  |  | S06_1968680 | 70,057 |
|  |  |  |  |  |  |  | S06_1968681 | 70,056 |
|  |  |  |  |  |  |  | S06_1970442 | 68,295 |
|  |  |  |  |  |  |  | S06_1970597 | 68,140 |
|  |  |  |  |  |  |  | S06_1970602 | 68,135 |
| MNPR | Os06g0138700 | 6 | 2,042,909 | 2,047,320 | - | Cyclin-like F-box domain containing protein | S06_1968653 | 74,256 |
|  |  |  |  |  |  |  | S06_1968680 | 74,229 |
|  |  |  |  |  |  |  | S06_1968681 | 74,228 |
|  |  |  |  |  |  |  | S06_1970442 | 72,467 |
|  |  |  |  |  |  |  | S06_1970597 | 72,312 |
|  |  |  |  |  |  |  | S06_1970602 | 72,307 |
| MNPR | Os06g0138900 | 6 | 2,049,319 | 2,050,655 | + | Conserved hypothetical protein | S06_1968653 | 80,666 |
|  |  |  |  |  |  |  | S06_1968680 | 80,639 |
|  |  |  |  |  |  |  | S06_1968681 | 80,638 |
|  |  |  |  |  |  |  | S06_1970442 | 78,877 |
|  |  |  |  |  |  |  | S06_1970597 | 78,722 |
|  |  |  |  |  |  |  | S06_1970602 | 78,717 |
| MNPR | Os06g0139000 | 6 | 2,056,222 | 2,061,224 | - | LRR-type F-box protein, Vegetative growth, Floral organ specification | S06_1968653 | 87,569 |
|  |  |  |  |  |  |  | S06_1968680 | 87,542 |
|  |  |  |  |  |  |  | S06_1968681 | 87,541 |
|  |  |  |  |  |  |  | S06_1970442 | 85,780 |
|  |  |  |  |  |  |  | S06_1970597 | 85,625 |
|  |  |  |  |  |  |  | S06_1970602 | 85,620 |
| MNPR | Os06g0139150 | 6 | 2,063,175 | 2,064,511 | + | Conserved hypothetical protein | S06_1968653 | 94,522 |
|  |  |  |  |  |  |  | S06_1968680 | 94,495 |
|  |  |  |  |  |  |  | S06_1968681 | 94,494 |
|  |  |  |  |  |  |  | S06_1970442 | 92,733 |
|  |  |  |  |  |  |  | S06_1970597 | 92,578 |
|  |  |  |  |  |  |  | S06_1970602 | 92,573 |
| MNPR | Os06g0139200 | 6 | 2,069,979 | 2,071,929 | - | Hypothetical conserved gene | S06_1970442 | 99,537 |
|  |  |  |  |  |  |  | S06_1970597 | 99,382 |
|  |  |  |  |  |  |  | S06_1970602 | 99,377 |
| MNPR | Os06g0142100 | 6 | 2,194,354 | 2,197,374 | + | Cyclin-like F-box domain containing protein | S06_2296852 | -99,478 |
| MNPR | Os06g0142200 | 6 | 2,198,362 | 2,199,467 | - | Early nodulin | S06_2296852 | -97,385 |
| MNPR | Os06g0142300 | 6 | 2,204,751 | 2,205,510 | - | Early nodulin 93 ENOD93 protein family protein | S06_2296852 | -91,342 |
| MNPR | Os06g0142350 | 6 | 2,207,763 | 2,208,557 | - | Similar to Early nodulin (Fragment) | S06_2296852 | -88,295 |
| MNPR | Os06g0142400 | 6 | 2,211,616 | 2,212,483 | - | Early nodulin | S06_2296852 | -84,369 |
|  |  |  |  |  |  |  | S06_2310856 | -98,373 |
| MNPR | Os06g0142500 | 6 | 2,229,034 | 2,232,001 | + | Similar to Wall-associated kinase 3 | S06_2296852 | -64,851 |
|  |  |  |  |  |  |  | S06_2310856 | -78,855 |
| MNPR | Os06g0142550 | 6 | 2,230,654 | 2,232,725 | - | Non-protein coding transcript | S06_2296852 | -64,127 |
|  |  |  |  |  |  |  | S06_2310856 | -78,131 |
| MNPR | Os06g0142600 | 6 | 2,233,157 | 2,235,761 | - | Similar to ELF3 protein | S06_2296852 | -61,091 |
|  |  |  |  |  |  |  | S06_2310856 | -75,095 |
| MNPR | Os06g0142650 | 6 | 2,240,754 | 2,242,139 | - | Similar to Avr9/Cf-9 rapidly elicited protein 11 (Fragment) | S06_2296852 | -54,713 |
|  |  |  |  |  |  |  | S06_2310856 | -68,717 |
| MNPR | Os06g0142625 | 6 | 2,240,589 | 2,242,176 | + | Hypothetical conserved gene | S06_2296852 | -54,676 |
|  |  |  |  |  |  |  | S06_2310856 | -68,680 |
| MNPR | Os06g0142700 | 6 | 2,242,777 | 2,245,693 | - | Cytochrome c oxidase, subunit Vb family protein | S06_2296852 | -51,159 |
|  |  |  |  |  |  |  | S06_2310856 | -65,163 |
| MNPR | Os06g0142800 | 6 | 2,246,874 | 2,249,836 | + | Ribosomal L11 methyltransferase domain containing protein | S06_2296852 | -47,016 |
|  |  |  |  |  |  |  | S06_2310856 | -61,020 |
| MNPR | Os06g0142900 | 6 | 2,250,032 | 2,255,627 | - | Transketolase, C-terminal/Pyruvate-ferredoxin oxidoreductase, domain II domain containing protein | S06_2296852 | -41,225 |
|  |  |  |  |  |  |  | S06_2310856 | -55,229 |
| MNPR | Os06g0143000 | 6 | 2,255,870 | 2,259,999 | + | Iron-superoxide dismutase | S06_2296852 | -36,853 |
|  |  |  |  |  |  |  | S06_2310856 | -50,857 |
| MNPR | Os06g0143100 | 6 | 2,260,682 | 2,262,973 | - | Similar to Cadmium tolerant 1 | S06_2296852 | -33,879 |
|  |  |  |  |  |  |  | S06_2310856 | -47,883 |
| MNPR | Os06g0143400 | 6 | 2,273,709 | 2,278,508 | + | Similar to Acyl-ACP thioesterase (Fragment) | S06_2296852 | -18,344 |
|  |  |  |  |  |  |  | S06_2310856 | -32,348 |
| MNPR | Os06g0143600 | 6 | 2,284,948 | 2,285,435 | - | Hypothetical protein | S06_2296852 | -11,417 |
|  |  |  |  |  |  |  | S06_2310856 | -25,421 |
| MNPR | Os06g0143700 | 6 | 2,291,665 | 2,297,803 | - | SULTR-like phosphorus distribution transporter, Control of the allocation of phosphorus to the grain | S06_2296852 | 0 |
|  |  |  |  |  |  |  | S06_2310856 | -13,053 |
| MNPR | Os06g0143750 | 6 | 2,292,079 | 2,297,384 | + | Hypothetical gene | S06_2296852 | 0 |
|  |  |  |  |  |  |  | S06_2310856 | -13,472 |
| MNPR | Os06g0143800 | 6 | 2,299,762 | 2,300,237 | - | Non-protein coding transcript | S06_2296852 | 2,910 |
|  |  |  |  |  |  |  | S06_2310856 | -10,619 |
| MNPR | Os06g0143900 | 6 | 2,319,838 | 2,328,652 | - | Similar to Coatomer protein complex, beta prime | S06_2296852 | 22,986 |
|  |  |  |  |  |  |  | S06_2310856 | 8,982 |
| MNPR | Os06g0143950 | 6 | 2,326,618 | 2,328,176 | + | Non-protein coding gene | S06_2296852 | 29,766 |
|  |  |  |  |  |  |  | S06_2310856 | 15,762 |
| MNPR | Os06g0144000 | 6 | 2,328,884 | 2,333,422 | + | BRCT domain containing protein | S06_2296852 | 32,032 |
|  |  |  |  |  |  |  | S06_2310856 | 18,028 |
| MNPR | Os06g0144200 | 6 | 2,335,435 | 2,337,189 | - | Similar to Pectate lyase homolog | S06_2296852 | 38,583 |
|  |  |  |  |  |  |  | S06_2310856 | 24,579 |
| MNPR | Os06g0144600 | 6 | 2,357,311 | 2,364,516 | - | Peptidase M14, carboxypeptidase A family protein | S06_2296852 | 60,459 |
|  |  |  |  |  |  |  | S06_2310856 | 46,455 |
| MNPR | Os06g0144800 | 6 | 2,365,747 | 2,371,092 | - | Similar to GTP-binding protein lepA | S06_2296852 | 68,895 |
|  |  |  |  |  |  |  | S06_2310856 | 54,891 |
| MNPR | Os06g0144900 | 6 | 2,371,993 | 2,373,947 | + | Similar to Pectate lyase homolog | S06_2296852 | 75,141 |
|  |  |  |  |  |  |  | S06_2310856 | 61,137 |
| MNPR | Os06g0145000 | 6 | 2,376,721 | 2,378,278 | + | Similar to Pectate lyase homolog | S06_2296852 | 79,869 |
|  |  |  |  |  |  |  | S06_2310856 | 65,865 |
| MNPR | Os06g0145200 | 6 | 2,379,596 | 2,381,937 | + | Transferase family protein | S06_2296852 | 82,744 |
|  |  |  |  |  |  |  | S06_2310856 | 68,740 |
| MNPR | Os06g0145600 | 6 | 2,393,822 | 2,395,563 | + | Transferase family protein | S06_2296852 | 96,970 |
|  |  |  |  |  |  |  | S06_2310856 | 82,966 |
| MNPR | Os06g0145700 | 6 | 2,394,969 | 2,400,012 | - | WRI1 homolog | S06_2296852 | 98,117 |
|  |  |  |  |  |  |  | S06_2310856 | 84,113 |
| MNPR | Os06g0145800 | 6 | 2,404,410 | 2,407,050 | + | Similar to Whirly family nucleic acid binding protein | S06_2310856 | 93,554 |
| MNPR | Os06g0145950 | 6 | 2,406,657 | 2,408,374 | - | Hypothetical conserved gene | S06_2310856 | 95,801 |
| MNPR | Os06g0146100 | 6 | 2,409,177 | 2,417,567 | + | NB-ARC domain containing protein | S06_2310856 | 98,321 |
| MNPR | Os06g0146900 | 6 | 2449211 | 2450670 | - | Hypothetical protein | S06_2550351 | -99681 |
| MNPR | Os06g0147000 | 6 | 2452482 | 2453047 | - | Conserved hypothetical protein | S06_2550351 | -97304 |
| MNPR | Os06g0147100 | 6 | 2456430 | 2457127 | - | Conserved hypothetical protein | S06_2550351 | -93224 |
| MNPR | Os06g0147200 | 6 | 2460059 | 2460868 | + | Conserved hypothetical protein | S06_2550351 | -89483 |
| MNPR | Os06g0147250 | 6 | 2461235 | 2461235 | + | Hypothetical conserved gene | S06_2550351 | -89116 |
| MNPR | Os06g0147300 | 6 | 2471739 | 2472345 | + | Conserved hypothetical protein | S06_2550351 | -78006 |
| MNPR | Os06g0147400 | 6 | 2476594 | 2477395 | - | Conserved hypothetical protein | S06_2550351 | -72956 |
| MNPR | Os06g0147600 | 6 | 2496635 | 2497203 | - | Conserved hypothetical protein | S06_2550351 | -53148 |
| MNPR | Os06g0147800 | 6 | 2497864 | 2501888 | - | Similar to Mitogen-activated protein kinase kinase 2 | S06_2550351 | -48463 |
| MNPR | Os06g0148200 | 6 | 2514767 | 2516856 | + | Lipase, GDSL domain containing protein | S06_2550351 | -33495 |
| MNPR | Os06g0148300 | 6 | 2517213 | 2519939 | + | Conserved hypothetical protein | S06_2550351 | -30412 |
| MNPR | Os06g0148500 | 6 | 2526015 | 2527305 | + | Cyclin-like F-box domain containing protein | S06_2550351 | -23046 |
| MNPR | Os06g0148600 | 6 | 2528104 | 2530646 | + | Cyclin-like F-box domain containing protein | S06_2550351 | -19705 |
| MNPR | Os06g0148700 | 6 | 2531619 | 2533591 | + | Cyclin-like F-box domain containing protein | S06_2550351 | -16760 |
| MNPR | Os06g0148800 | 6 | 2534419 | 2535630 | + | Putative Cyclin-like F-box | S06_2550351 | -14721 |
| MNPR | Os06g0148900 | 6 | 2536904 | 2538469 | + | Cyclin-like F-box domain containing protein | S06_2550351 | -11882 |
| MNPR | Os06g0149000 | 6 | 2541031 | 2542197 | + | Putative Cyclin-like F-box | S06_2550351 | -8154 |
| MNPR | Os06g0149100 | 6 | 2545953 | 2548252 | + | Similar to Proline-rich protein APG-like | S06_2550351 | -2099 |
| MNPR | Os06g0149300 | 6 | 2554636 | 2556664 | - | Conserved hypothetical protein | S06_2550351 | 4285 |
| MNPR | Os06g0149400 | 6 | 2557018 | 2561217 | + | Similar to Chitinase A | S06_2550351 | 6667 |
| MNPR | Os06g0149450 | 6 | 2557440 | 2560980 | - | Hypothetical gene | S06_2550351 | 7089 |
| MNPR | Os06g0149500 | 6 | 2564187 | 2566639 | + | Conserved hypothetical protein | S06_2550351 | 13836 |
| MNPR | Os06g0149700 | 6 | 2573180 | 2575677 | + | Cysteine synthase | S06_2550351 | 22829 |
| MNPR | Os06g0149801 | 6 | 2573233 | 2575717 | - | Hypothetical gene | S06_2550351 | 22882 |
| MNPR | Os06g0149900 | 6 | 2578090 | 2580721 | + | Similar to Cysteine synthase | S06_2550351 | 27739 |
| MNPR | Os06g0150100 | 6 | 2583172 | 2585157 | - | Conserved hypothetical protein | S06_2550351 | 32821 |
| MNPR | Os06g0150300 | 6 | 2586931 | 2587897 | - | Conserved hypothetical protein | S06_2550351 | 36580 |
| MNPR | Os06g0150400 | 6 | 2588060 | 2591249 | - | Conserved hypothetical protein | S06_2550351 | 37709 |
| MNPR | Os06g0150500 | 6 | 2591556 | 2593287 | - | Inorganic pyrophosphatase domain containing protein | S06_2550351 | 41205 |
| MNPR | Os06g0150600 | 6 | 2591801 | 2593428 | + | Transferase family protein | S06_2550351 | 41450 |
| MNPR | Os06g0151100 | 6 | 2632740 | 2634177 | - | Similar to Transferase | S06_2550351 | 82389 |
| MNPR | Os06g0151200 | 6 | 2634909 | 2640270 | - | Nucleotide-binding, alpha-beta plait domain containing protein | S06_2550351 | 84558 |
| MNPR | Os06g0151300 | 6 | 2641360 | 2644173 | - | Sybindin-like protein family protein | S06_2550351 | 91009 |
| MNPR | Os06g0151401 | 6 | 2643905 | 2645002 | + | Hypothetical gene | S06_2550351 | 93554 |
| DDTH | Os11g0670100 | 11 | 29,277,813 | 29,280,510 | + | Similar to Protein kinase domain containing protein, expressed | S11_29358169 | -77,659 |
| DDTH | Os11g0670700 | 11 | 29,290,252 | 29,307,255 | + | Similar to EAP30 family protein | S11_29358169 | -50,914 |
| DDTH | Os11g0670800 | 11 | 29,307,472 | 29,308,713 | + | Similar to EAP30 family protein | S11_29358169 | -49,456 |
| DDTH | Os11g0670900 | 11 | 29,311,531 | 29,312,399 | + | Hypothetical protein | S11_29358169 | -45,770 |
| DDTH | Os11g0671000 | 11 | 29,316,094 | 29,317,588 | - | Similar to Dormancy-associated protein | S11_29358169 | -40,581 |
| DDTH | Os11g0671100 | 11 | 29,316,369 | 29,317,266 | + | Conserved hypothetical protein | S11_29358169 | -40,903 |
| DDTH | Os11g0671600 | 11 | 29,331,968 | 29,338,304 | - | Hypothetical conserved gene | S11_29358169 | -19,865 |
| DDTH | Os11g0671650 | 11 | 29,341,783 | 29,345,477 | - | Hypothetical gene | S11_29358169 | -12,692 |
| DDTH | Os11g0672200 | 11 | 29,352,162 | 29,352,769 | + | Similar to Protein kinase domain containing protein, expressed | S11_29358169 | -5,400 |
| DDTH | Os11g0672300 | 11 | 29,354,934 | 29,357,281 | + | Similar to Protein kinase domain containing protein, expressed | S11_29358169 | -888 |
| DDTH | Os11g0672400 | 11 | 29,358,621 | 29,364,907 | - | Kinesin, motor region domain containing protein; Calponin homology domain containing protein | S11_29358169 | 452 |
| DDTH | Os11g0672600 | 11 | 29,374,669 | 29,377,511 | + | Reverse transcriptase domain containing protein | S11_29358169 | 16,500 |
| DDTH | Os11g0672700 | 11 | 29,378,911 | 29,383,614 | - | Transgene silencing.; RecQ DNA helicase | S11_29358169 | 20,742 |
| DDTH | Os11g0672800 | 11 | 29,390,611 | 29,395,078 | - | Conserved hypothetical protein | S11_29358169 | 32,442 |
| DDTH | Os11g0672900 | 11 | 29,390,841 | 29,392,140 | + | Protein of unknown function DUF1645 family protein | S11_29358169 | 32,672 |
| DDTH | Os11g0673100 | 11 | 29,399,968 | 29,402,492 | - | Seed maturation protein, osa-miR164c target gene, Regulation of seed vigor | S11_29358169 | 41,799 |
| DDTH | Os11g0673150 | 11 | 29,400,095 | 29,402,373 | + | Hypothetical gene | S11_29358169 | 41,926 |
| DDTH | Os11g0673200 | 11 | 29,403,281 | 29,410,162 | - | Similar to Auxin-induced beta-glucosidase | S11_29358169 | 45,112 |
| DDTH | Os11g0673400 | 11 | 29,409,610 | 29,410,203 | + | Hypothetical gene | S11_29358169 | 51,441 |
| DDTH | Os11g0673600 | 11 | 29,421,443 | 29,424,544 | + | Similar to NB-ARC domain containing protein, expressed | S11_29358169 | 63,274 |
| DDTH | Os11g0676100 | 11 | 29,441,658 | 29,442,932 | + | Similar to NB-ARC domain containing protein, expressed | S11_29358169 | 83,489 |
