# Supplementary material for: Phenotypic variation and genome-wide association studies of main culm panicle node number, maximum node production rate, and degree-days to heading in rice
Source: BMC Genomics. 2022 May 23;23:390. doi: 10.1186/s12864-022-08629-y (PMC9125873; doi:10.1186/s12864-022-08629-y)
Supplement: Supplementary file 3 — Additional File 3. Appendix 1. Phenotyping for Maximum Node Production Rate (MNPR) and Main Culm Panicle Node Number (MCPNN) [file 12864_2022_8629_MOESM3_ESM.docx]

**Supplementary Information**

**Appendix 1. Phenotyping for Maximum Node Production Rate (MNPR) and Main Culm Panicle Node Number (MCPNN)**

For each rice accession, one meter within a row was designated for collecting the leaf count data used to estimate MCPNN and MNPR. This one-meter length was delimited by a marking flag (Presco Safety Marking Products, Sherman, TX) on each end. Starting at the third leaf stage, the number of emerged leaves on the main culm were recorded, and the last fully expanded leaf was marked with a permanent marker. Every week, newly-emerged leaves after the marked leaf from the previous observation were counted, with the last fully expanded leaf marked as a reference for the succeeding observation. The main culms were tagged with labeled shipping tags (Uline, Pleasant Prairie, WI) to distinguish them from tillers.

As each node gives rise to a leaf, MNPR was estimated through regression of the average leaf emergence data during the 3^rd^ to 7^th^ leaf stages with cumulative degree-days > 10℃ after planting. Leaf counting was continued until heading in order to estimate the MCPNN, which is the number of leaves on the main culm plus one for its panicle (Samonte et al., 2006).

**Reference:**

Samonte SOPB, Wilson LT, Tabien RE. Maximum node production rate and main culm node number contributions to yield and yield-related traits in rice. Field Crops Research. 2006 Apr 30;96(2–3):313–319.
